# Virome Profiling via sRNA-Seq and Sample-Level Coding-Region Diversity of Tomato Brown Rugose Fruit Virus in Protected Tomato Crops in Xinjiang

**DOI:** 10.3390/v18101127

**Published:** 2026-10-11

**Authors:** Jiecai Li, Xin Huang, Wenfang Luo, Junhui Zhou, Xiang Zhang, Siqiong Tang, Wei He, Jianjun Xu

**Affiliations:** 1 Institute of Plant Protection, Xinjiang Uygur Autonomous Region Academy of Agricultural Sciences, Key Laboratory of Integrated Pest Management on Crops in Northwestern Oasis, Ministry of Agriculture and Rural Affairs, Xinjiang Key Laboratory of Agricultural Bio-Safety, Urumqi 830091, China; l13099847029@126.com (J.L.); huangxin0924@126.com (X.H.); lf576263465@163.com (W.L.); junhuiqzhou@163.com (J.Z.); kczx268@126.com (X.Z.); shzutangsq@163.com (S.T.); 2 College of Agronomy, Xinjiang Agricultural University, Urumqi 830091, China

**Keywords:** tomato brown rugose fruit virus, small-RNA sequencing, mixed infection, genomic surveillance, Xinjiang

## Abstract

Viral diseases threaten protected tomato production in Xinjiang, China, yet the virus spectrum in symptomatic crops and the local sequence context of Tomato brown rugose fruit virus (ToBRFV) remain incompletely characterized. We used pooled small-RNA sequencing for virus discovery and individual reverse transcription PCR (RT-PCR)/Sanger confirmation in 116 symptomatic tomato samples and compared nine Xinjiang ToBRFV coding-region genomes with 435 curated public GenBank sequences. Nine viruses were identified in the symptomatic tomato samples, with ToBRFV being the most frequently detected (113/116, 97.4%). Virus co-detections occurred in 115 samples (99.1%), with four-virus combinations being the most common. In the 444-sequence ToBRFV coding-region dataset, the nine Xinjiang genomes obtained in this study represented distinct exact coding-sequence types and occupied multiple phylogenetic placements; 139 biallelic segregating sites were observed among them. These results document complex multi-virus co-detection in symptomatic protected-tomato samples and provide a local ToBRFV sequence resource. Because sampling was symptom-targeted and the genome set was small, representative sampling and epidemiological metadata are required to estimate regional prevalence, ToBRFV diversity and transmission history.

## 1. Introduction

Tomato (*Solanum lycopersicum*) is an important vegetable crop in Xinjiang, where protected cultivation benefit from intense solar radiation, large diurnal temperature differences and low humidity [1]. Viral diseases have become a persistent constraint on protected-tomato production, affecting yield, fruit quality and seedling movement [2]. Because infected greenhouse tomatoes often show overlapping symptoms, including mosaic, chlorosis, leaf deformation and fruit rugosity, visual diagnosis alone cannot resolve the virus species present in symptomatic plants or distinguish single-virus detections from virus co-detections, an issue that is especially important when mixed infections can influence virus accumulation and symptom expression [3,4].

Among tomato-infecting viruses, Tomato brown rugose fruit virus (ToBRFV) is a quarantine tobamovirus of particular concern. It causes severe foliar and fruit symptoms, is readily transmitted mechanically and through contaminated production materials, and has been reported to infect tomato cultivars carrying the widely used *Tm-2^2^* resistance gene [5,6,7,8]. These features make ToBRFV a priority target for molecular surveillance, but sequence variants detected in field isolates should be interpreted as surveillance markers unless they are linked to phenotypic or mechanistic evidence.

Previous Chinese and regional surveys have reported multiple tomato-infecting viruses, including Tomato yellow leaf curl virus (TYLCV), Tomato chlorosis virus (ToCV), Cucumber mosaic virus (CMV), Tobacco mosaic virus (TMV), Tomato mosaic virus (ToMV), Tomato mottle mosaic virus (ToMMV), Southern tomato virus (STV) and ToBRFV [9,10,11,12]. In Xinjiang, prior records include ToBRFV, TYLCV, STV, ToMV and CMV, and recent work has established important occurrence information for protected-tomato systems, particularly in southern Xinjiang [10,12]. However, the connection between pool-level virus discovery, sample-level confirmation and coding-region variation in local ToBRFV genomes remains insufficiently resolved. Public ToBRFV comparisons have also shown limited overall divergence, underscoring the need to interpret local genomes within a carefully curated public sequence context [13].

Here, we combined pooled small-RNA sequencing (sRNA-seq) with individual reverse transcription PCR (RT-PCR)/Sanger confirmation to identify viruses and describe virus co-detection patterns in 116 symptomatic tomato samples from protected cultivation across Xinjiang. We then amplified and sequenced ToBRFV coding-region sequences from nine samples and analyzed them alongside curated public GenBank sequences. The study was designed to provide an integrated local surveillance dataset and to place sampled Xinjiang ToBRFV coding-region sequences within a curated public sequence context; it was not designed to estimate regional prevalence, sequence-type frequencies, introduction routes or transmission history.

## 2. Materials and Methods

### 2.1. Sample Collection and Composite-Pool Preparation

**Figure 1:**
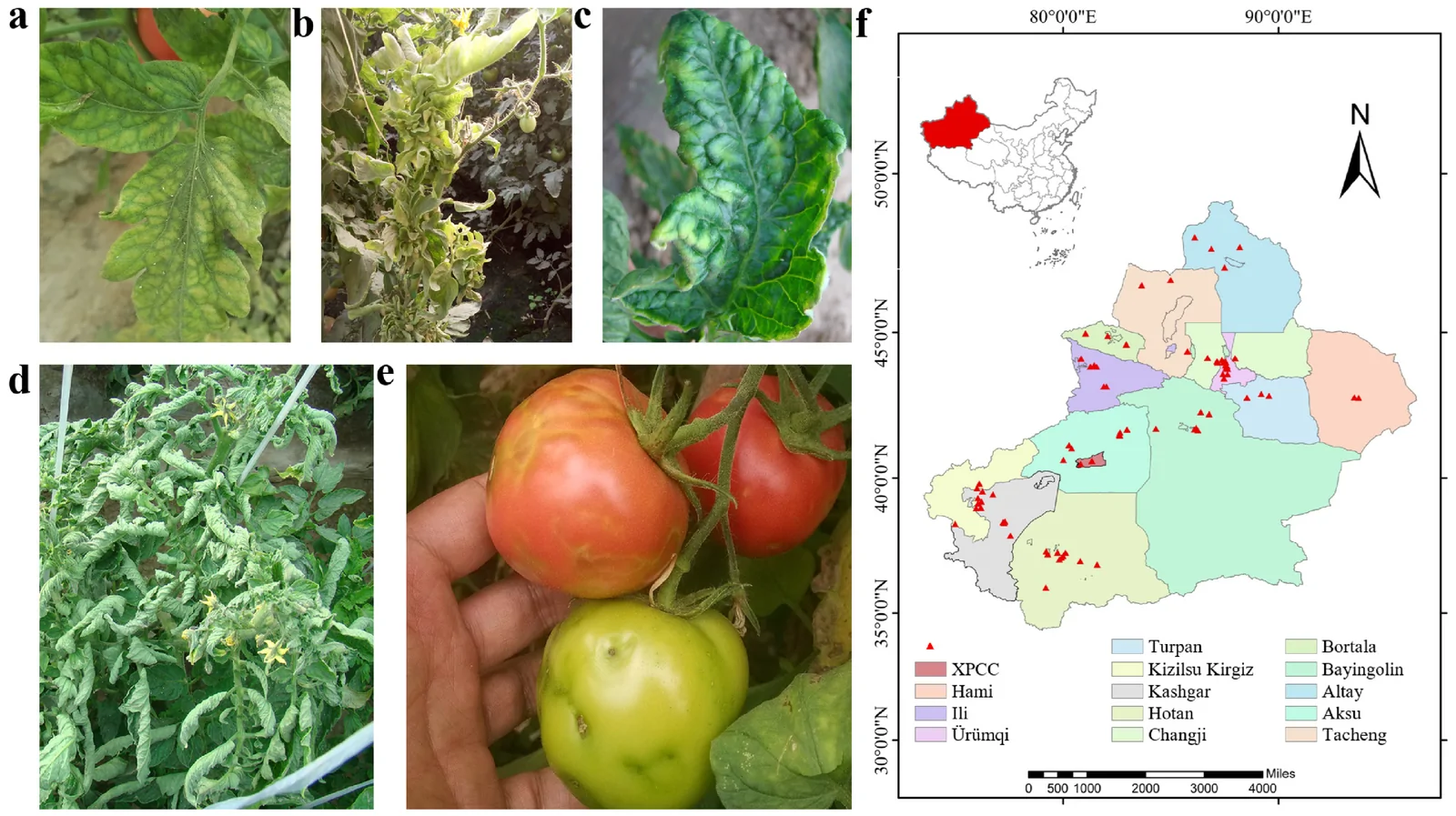
Representative disease symptoms and sampling locations of protected tomato crops in Xinjiang, China. (**a**–**e**) Representative foliar and fruit symptoms observed in the sampled symptomatic plants, including mosaic or mottling, chlorosis, leaf blistering/epinasty, leaf deformation and brown rugose fruit lesions. (**f**) Geographical distribution of tomato sampling sites across the Xinjiang Uygur Autonomous Region, China. Samples were collected from 14 locations, covering southern and northern Xinjiang. The map was generated using ArcGIS v10.8.

Field surveys were conducted during the 2024 and 2025 growing seasons at 14 protected-tomato production locations across the Xinjiang Uygur Autonomous Region, China (Figure 1f). A total of 116 symptomatic leaf and fruit samples were collected from individually identified plants showing mosaic, chlorosis, leaf blistering or epinasty, leaf deformation, or brown rugose fruit lesions (Figure 1a–e). Each sample was labelled separately, transported on dry ice, and stored at −80 °C until processing.

The 116 individual samples were assigned to 17 composite pools according to geographic origin (Table S1), with equal masses of tissue from each individual sample combined to generate each composite pool before RNA extraction. Locations with larger sample numbers, including Hotan, Kashgar and Bayingolin, were divided into two pools to keep the total amount of pooled tissue compatible with the extraction procedure. Pool identity was kept distinct from individual-sample identity throughout the study, and the original individual samples were retained separately for sample-level RT-PCR confirmation.

### 2.2. RNA Extraction, Small-RNA Sequencing and Virus Discovery

Approximately 100 mg of pooled leaf tissue from each of the 17 composite samples was used for total RNA extraction with the Spin Plant RNA Mini Kit (Invitek, Berlin, Germany). RNA purity, concentration and integrity were assessed using a NanoDrop spectrophotometer (Thermo Fisher Scientific, Wilmington, DE, USA), Qubit 2.0 fluorometer (Invitrogen, Carlsbad, CA, USA) and Agilent 2100 Bioanalyzer (Agilent Technologies, Santa Clara, CA, USA), respectively. Only RNA meeting the library-acceptance criteria was used for sequencing (RNA concentration ≥ 100 ng·µL^−1^; total RNA ≥ 1 µg; *A*260/*A*280 = 1.9–2.1; *A*260/*A*230 > 1.7; RIN ≥ 8.0). Small-RNA libraries were prepared by adapter ligation, reverse transcription, PAGE-based size selection and PCR enrichment, and were sequenced as single-end 50 nt reads on an Illumina NovaSeq platform (Illumina, San Diego, CA, USA) by Nanjing Jisihuiyuan Biotechnology Co., Ltd. (Nanjing, Jiangsu, China).

Raw reads were quality-filtered with fastp v0.20.0 (-q 5 -n 5) and trimmed with cutadapt v1.18 (-a AGATCGGAAGAGCACACGTCT -O 3 -m 18 -M 30 -f fastq -q 20). Clean reads were analyzed with a VirusDetect-based workflow. Reads were aligned to the VirusDetect v248 viral sequence database (database files dated 14 April 2022, http://bioinfo.bti.cornell.edu/ftp/program/VirusDetect/virus_database/v248/) using BWA v0.7.17-r1188 with default settings reported in the provider methods file. In parallel, reads were aligned to the tomato reference genome GCA_000188115.4, and unmapped reads were assembled with Velvet v1.1.07 using default settings reported in the provider methods file. Non-redundant non-host contigs were searched against viral nucleotide and protein databases using megablast/blastall v2.2.16 [14]. Virus detections were accepted when the corresponding candidate path met the predefined support thresholds: BLASTN *E*-value ≤ 1 × 10^−5^, BLASTX *E*-value ≤ 1 × 10^−2^, nucleotide identity ≥ 60%, contig length > 40 nt or ≥100 nt for BLASTX candidates, genome coverage > 10% and ≥1 supporting contig. Virus-aligned read counts and percentages were summarized from the delivered mapping reports, and pool-level small-RNA size distributions were calculated from the cutadapt length-distribution summaries.

### 2.3. Individual-Sample Virus Confirmation and Co-Detection Summaries

To confirm the viruses detected by pooled sRNA sequencing at the individual-plant level, total RNA was extracted separately from each of the 116 retained symptomatic samples and used for RT-PCR. Each sample was screened for all nine viruses using virus-specific primer pairs that were either designed with Primer Premier 5 or adopted from published studies (Table S2). First-strand cDNA was synthesized from 1 µg of total RNA with the HiScript IV 1st Strand cDNA Synthesis Kit (Vazyme, Nanjing, Jiangsu, China). Each 25 µL PCR contained 1 µL cDNA, 12.5 µL 2× Taq Master Mix (Vazyme, Nanjing, Jiangsu, China), 1 µL each of forward and reverse primers (10 µM), and 9.5 µL nuclease-free water. Cycling took place at 94 °C for 4 min; 35 cycles were performed at 94 °C for 30 s, at 58 °C for 30 s, and at 72 °C for up to 90 s, depending on amplicon length; then at 72 °C for 5 min. Each PCR batch included a no-template control. For each of the nine virus-specific assays, a plasmid template known to yield the expected target amplicon was used as a positive amplification control. Expected-size amplicons from the nine virus assays were purified and sequenced by Sangon Biotech (Shanghai) Co., Ltd. (Shanghai, China), with virus identity confirmed by BLASTn against NCBI nt (≥95% nucleotide identity and ≥90% query coverage).

Final virus calls for individual samples were based on RT-PCR/Sanger confirmation, irrespective of whether the corresponding pool-level sRNA-seq result was concordant. For each virus, the observed detection proportion was calculated as the number of confirmed positive samples among the 116 symptomatic samples tested. Virus detections were then summarized by sampling origin, number of viruses per sample, exact virus combinations, and pairwise co-detections. As sampling was symptom-based, these summaries were descriptive and were not used to estimate regional prevalence or population-level infection frequency.

### 2.4. Overlapping RT-PCR and Consensus Assembly of Xinjiang ToBRFV Isolates

The nine field samples used for ToBRFV genome sequencing were selected purposively, taking into account their geographic distribution across Xinjiang and the importance of the sampled regions for protected-tomato production. The selected set included one sample each from Aksu, Kashgar, Yili, Kizilsu and Urumqi, and two each from Hotan and Turpan. Each genome was amplified as six overlapping RT-PCR fragments with the ToBRFV-specific primer pairs listed in Table S3. Total RNA extraction and first-strand cDNA synthesis followed the procedures described above. Amplicons were sequenced by Sangon Biotech (Shanghai) Co., Ltd. (Shanghai, China), and reads from overlapping amplicons were trimmed and assembled into consensus sequences. Each consensus was aligned to NC_028478.1 to verify sequence orientation and the completeness and continuity of the regions encoding the replicase, movement protein (MP) and coat protein (CP). Open-reading frames (ORF) were predicted with NCBI ORFfinder and checked by translation against the corresponding reference annotations. Final consensus sequences were deposited in GenBank (PQ774335 and PZ906282–PZ906289). Data S1 links these accessions to the corresponding Xinjiang isolate names, local analysis identifiers, sample identifiers and sRNA-seq composite-library identifiers.

### 2.5. Publicly Available ToBRFV Sequence Retrieval and Metadata Curation

Public ToBRFV records were curated from GenBank records retrieved on 19 August 2026. The main matrix retained accessions with complete annotated replicase readthrough, *MP* and *CP* coding features after applying predefined quality and provenance filters. Metadata curation, inclusion/exclusion records and duplicate-resolution decisions are provided in Methods S1 and Data S1.

### 2.6. Coding-Region Alignment, Sequence-Type Resolution and Contextual Panel Construction

The curated public sequences and nine Xinjiang genomes were projected to the NC_028478.1 coding coordinates to produce a 6129 nt fixed coding-region alignment comprising replicase, *MP* and *CP*. This alignment was used for phylogenetic, distance and diversity analyses, with region-specific quality-control summaries retaining information on insertion, deletion and callable-site handling. The gap-free 85-sequence core used for recombination screening was derived from this curated coding matrix as described in Methods S1.

Exact aligned coding-sequence types were defined from the gap-aware coding-region alignment. A reduced contextual panel was then constructed for tree visualization and sensitivity analyses by retaining all nine Xinjiang genomes, all other public Chinese sequences and NC_028478.1, and by selecting additional representatives within country–year strata. Panel selection, retention metrics, random seeds and replicate-panel construction are detailed in Methods S1. Variants were annotated relative to NC_028478.1 and summarized by coding region, including predicted codon effects for descriptive maps.

### 2.7. Phylogenetic and Genetic-Diversity Analyses

Maximum-likelihood trees were inferred with IQ-TREE v3.0.1 from the full coding-region matrix, the primary reduced contextual panel and 100 replicate reduced contextual panels, with ModelFinder model selection and ultrafast-bootstrap (UFBoot) and Shimodaira–Hasegawa-like approximate likelihood-ratio test (SH-like aLRT) branch support. The robustness of Xinjiang isolate placements was evaluated across 100 replicate reduced-panel trees generated with alternative public-sequence backgrounds. A second sensitivity analysis specifically varied non-key Chinese background sequences while retaining the nine Xinjiang genomes, key Chinese reference sequences associated with focal placements, and the non-Chinese background. The temporal signal was assessed using root-to-tip regression under five dated-sequence filtering strategies.

Pairwise *p*-distances were calculated from positions that were callable in each sequence pair. For standardized public comparisons, the nine Xinjiang genomes were compared with eligible country groups from the primary reduced public background by repeated eight-record subsampling, and within-group distances and a descriptive between-group distance contrast were summarized across draws. Relationships between exact coding-sequence types were displayed as a minimum-spanning network, and principal-coordinate analysis (PCoA) was performed on the full pairwise *p*-distance matrix.

Coding-region genetic diversity was quantified using DnaSP v6.12.01 [15] from record-level multiple sequence alignments that preserved identical sequences and removed columns containing gaps or missing data for each analyzed matrix. Standard diversity and neutrality metrics were computed across the full matrix, reduced contextual panel, eligible public groups, and nine Xinjiang ToBRFV genomes. Sliding-window estimates of π were generated with a window size of 300 nt and a step size of 100 nt. Methods S2 documents full analytical parameters, including IQ-TREE configurations, random seeds, replicate-panel assembly, distance and ordination workflows, DnaSP input specifications, group eligibility criteria, and definitions for temporal-signal filtering, alongside descriptions of supplementary sequence annotations deposited in Data S2.

### 2.8. Recombination Screening

Recombination was screened on the gap-free 85-sequence core alignment using PhiPack v1.0 [16]. The pairwise homoplasy index (PHI) and neighbour similarity score (NSS) tests were used as global recombination screens with 1000 permutations. Localized signals were evaluated with the PhiPack MaxChi scan and Profile sliding-window PHI analysis (500 nt window; 100 nt step). Four-gamete incompatibility counts and the completed RDP5 v5.93 sensitivity run were used to perform additional checks for screen-positive signals. Recombination support required corroboration by independent tests or consistent local-window support; isolated single-test signals were retained as sensitivity flags and excluded from parental-lineage, breakpoint and recombinant-exclusion inference. Positions reported for this screen refer to the gap-free core alignment. As a sensitivity check on the single-tree-history premise outside the 85-sequence core, exact-type-collapsed full-matrix and primary reduced-panel alignments were also screened with the same PHI/NSS/Profile/MaxChi logic; these expanded screens were interpreted as supplementary sensitivity checks rather than breakpoint or parental-lineage analyses.

### 2.9. Codon-Based Selection Analyses

Region-specific codon alignments were prepared from the primary reduced contextual panel for the 126 kDa replicase coding region, the full 183 kDa readthrough replicase coding region, and the MP and CP coding regions. The 183 kDa alignment included the N-terminal sequence shared with the 126 kDa replicase. Sequences containing ambiguous codons or unexpected ORF-disrupting changes were excluded from the corresponding regional datasets. Identical regional sequences were collapsed to one representative for the primary codeml analyses, with accession mappings linking representatives to the original records. Regional coordinates, translation checks, canonical stop-codon handling, exclusion criteria and deduplication procedures are detailed in Methods S3.

Site-model analyses used codeml in PAML v4.10.10 [17] with region-specific neighbour-joining user trees and fixed topology. Residues detected by models allowing ω > 1 were classified as sequence-based candidates rather than functional or adaptive sites. Bayes empirical Bayes (BEB) posterior probabilities and fixed-effects likelihood (FEL) *p* values were treated as distinct evidence measures, and discordant sensitivity results were used to downgrade the interpretation. CP and MP sensitivity analyses retained all eligible records, using codeml in PAML v4.10.10 for CP and FEL in HyPhy v2.5.101 for MP. Control settings, duplicate-handling rationale and diagnostic-run definitions are documented in Methods S3.

### 2.10. Sequence Composition and Feature Annotation

Selected previously reported MP residues, the replicase readthrough signal and putative subgenomic promoter-proximal windows were compared with the reference-aligned sequences as sequence-based annotations. Coordinate-based summaries used the 6129 nt reference coding projection: the 126 kDa readthrough stop codon occupied positions 3349–3351, the downstream 8 nt motif occupied positions 3352–3359, and the *MP*- and *CP*-proximal windows were 71-position intervals spanning the 70 nt immediately upstream of each start codon plus the first start-codon nucleotide (positions 4779–4849 and 5580–5650). These windows were treated as putative promoter-proximal sequence regions by positional analogy to mapped and comparative tobamovirus subgenomic promoter regions [18,19,20]. Variant density was counted across complete-call columns of the 444-sequence matrix. CpG and UpA observed/expected ratios and relative synonymous codon usage (RSCU) were summarized by coding region. Compositional summaries were compared between the Xinjiang and public sequences; RSCU profiles were also compared between host groups recorded as tomato and pepper. These analyses report sequence composition and annotation; functional effects require experimental tests.

## 3. Results

### 3.1. Virus Detection and Co-Detection in Symptomatic Tomato Samples

Sequencing of the 17 composite pools generated 11.9–104.8 million raw reads per pool (median, 19.2 million), of which 11.1–94.1% were retained after trimming and filtering. Viral-reference searches identified 3076 unique contigs (Table S1). Virus-aligned reads accounted for 3.28–71.29% of processed reads (median, 16.44%). Pool-level clean-read size summaries showed a predominant 22 nt class in 11 pools, a 21 nt class in two and a 24 nt class in four (Figure S1).

**Figure 2:**
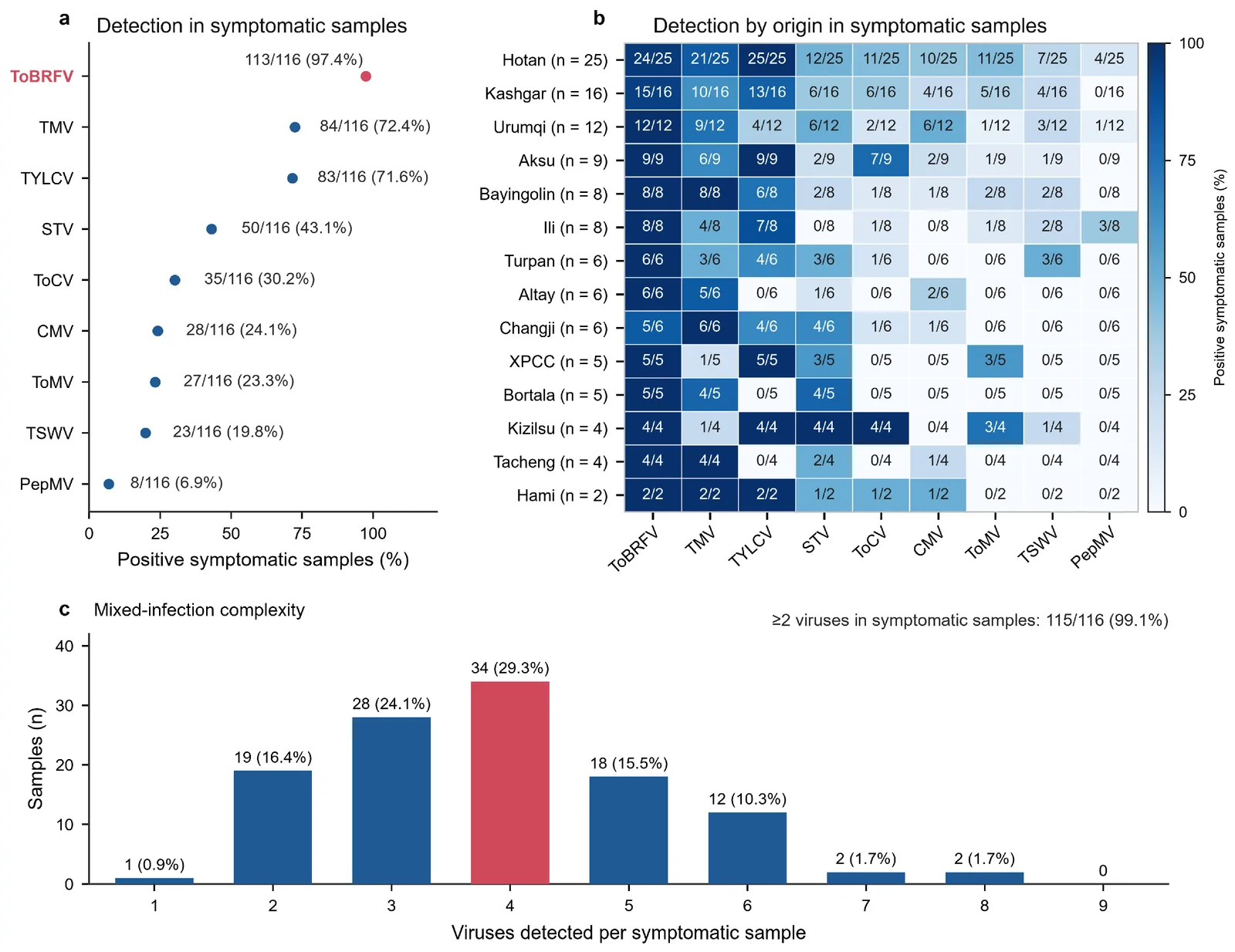
RT-PCR/Sanger-confirmed virus detections in symptomatic tomato samples (*n* = 116). (**a**) Points show observed detection proportions; labels give positive/total samples and percentages. (**b**) Detections by sampling origin. Cells show positive/total samples, colours indicate detection percentages and row labels give sample sizes. (**c**) Numbers of virus species detected per sample; bars and labels show counts and percentages. Red highlights ToBRFV in a and the modal four-virus category in c. The sampling-origin summaries are descriptive. Kizilsu, Kizilsu Kirgiz Autonomous Prefecture; XPCC, Xinjiang Production and Construction Corps.

Similarity searches identified nine virus species across the 17 pools: ToBRFV, TYLCV, ToMV, STV, TMV, ToCV, Tomato spotted wilt virus (TSWV), Pepino mosaic virus (PepMV) and CMV. ToBRFV had the highest observed detection proportion among the 116 symptomatic samples, with 113/116 samples (97.41%), followed by TMV (84/116, 72.41%) and TYLCV (83/116, 71.55%). Detection frequencies for the remaining viruses ranged from 6.90% (PepMV) to 43.10% (STV) (Figure 2a). Across the nine RT-PCR assays, 451 expected-size positive amplicons were obtained, and all 451 were subjected to bidirectional Sanger sequencing and BLASTn confirmation. Pool-level detections and individual-sample confirmations are summarized in Table S4. The complete virus detection matrix and associated sample-level metadata are provided in Data S3.

Within this symptomatic sample set, co-detections were observed in 115 samples (99.14%), with a mean of 3.89 viruses per sample, and four-virus combinations were the most frequent category (34/116, 29.31%; Figure 2c). Forty-seven exact virus-combination profiles were observed; the ten most frequent profiles accounted for 63/116 samples (54.31%), led by ToBRFV+TMV (10/116), ToBRFV+TMV+TYLCV+ToMV+TSWV (8/116), and ToBRFV+TMV+STV, ToBRFV+TMV+TYLCV+STV and ToBRFV+TMV+TYLCV+STV+ToCV+CMV (7/116 each; Table S5). Pairwise co-detections were most frequent for ToBRFV-TMV (82/116), ToBRFV-TYLCV (80/116) and TMV-TYLCV (55/116), and ToBRFV was accompanied by TMV and/or TYLCV in 109/116 samples (93.97%).

Virus detections were summarized by sampling origin, with unequal numbers of symptomatic plants across groups. The mean number of viruses detected per plant was 4.45 among symptomatic plants sampled from southern Xinjiang and 3.24 among those sampled from northern Xinjiang. Across the sampled plants from Hotan, all nine target viruses were detected collectively, with a mean of 5.0 viruses per plant. Plants sampled from Kizilsu had the largest observed mean (5.25), whereas lower means were recorded among plants sampled from Altay, Bortala and Tacheng (Table S6; Figure 2b).

### 3.2. Sample-Level Coding-Region Diversity and Phylogenetic Placement of Xinjiang ToBRFV Genomes

To place the ToBRFV detections in genomic context, we analyzed nine ToBRFV coding-region consensus genomes obtained from protected-tomato samples collected from different regions of Xinjiang. For public comparison, 435 curated ToBRFV sequences were retained from 482 GenBank records screened, with the remaining 47 records excluded or resolved as duplicates. Together with the nine Xinjiang genomes, the final dataset comprised 444 sequences in a 6129 nt coding-region matrix, of which 6113 positions contained unambiguous, non-gap nucleotides in all sequences. Known public collection years spanned 2014–2025. Netherlands records accounted for 216/435 publicly available sequences (49.7%).

The alignment contained 344 exact coding-sequence types among 444 records, reflecting strict identity across the 6129 nt coding-region matrix. The full matrix contained 1094 variable sites, comprising 1012 biallelic and 82 multiallelic sites, and DnaSP estimates for the full matrix were *h* = 344, *Hd* = 0.9953, *π* = 0.00354 and *k* = 21.619. The reduced contextual panel had *h* = 273, *Hd* = 0.9992, *π* = 0.00385 and *k* = 23.507. For the nine sampled Xinjiang genomes, all records were distinct (*h* = 9, *Hd* = 1.000) and the descriptive nucleotide diversity was *π* = 0.00626 (*k* = 38.389). These values describe this nine-genome set and were not treated as estimates of Xinjiang-wide sequence-type frequency or population diversity.

Each sampled Xinjiang genome was a unique exact coding-sequence type within the curated dataset, and the minimum-spanning network retained a star-like central structure (Figure S2). The nine Xinjiang sequences contained 139 biallelic segregating sites.

**Figure 3:**
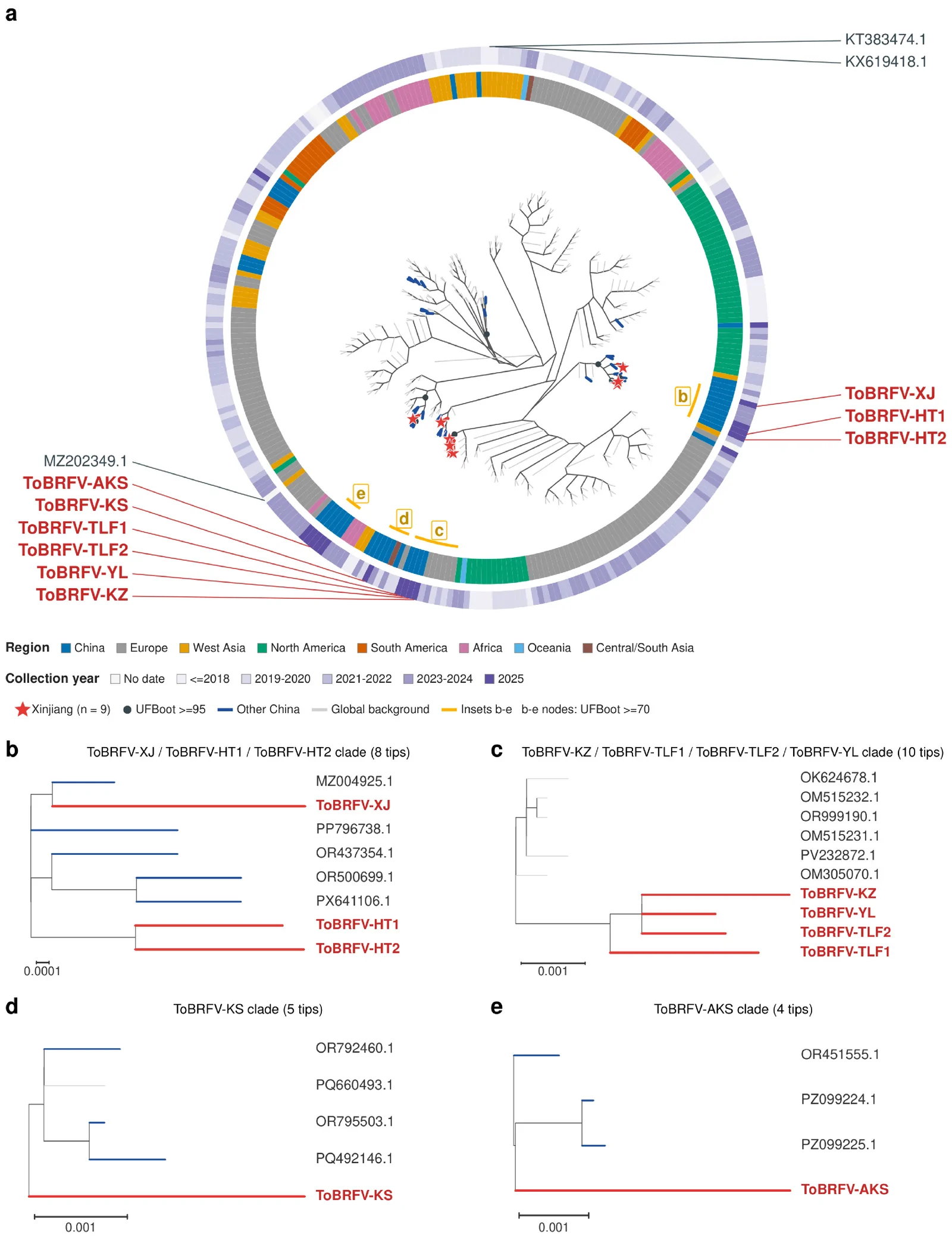
Contextual maximum-likelihood relationships of ToBRFV coding-region sequences. (**a**) Circular overview of the primary 300-sequence reduced contextual panel inferred from the 6129 nt concatenated replicase–MP–CP alignment using IQ-TREE 3.0.1 under TVM+F+I+R3 with 1000 UFBoot and 1000 SH-aLRT replicates. Branch lengths are not drawn to scale, and the orange b–e markers identify the clades enlarged in the corresponding panels. (**b**–**e**) Enlarged views of the clades marked in (**a**), with branch lengths drawn to scale and scale bars indicating substitutions per site. Xinjiang isolates are highlighted in red and other Chinese records in blue. Nodes with UFBoot ≥ 95 are marked; numerical SH-aLRT/UFBoot values for focal placements are reported in the text. Branch relationships are interpreted as sequence relationships; source, route and direction require additional epidemiological evidence. Xinjiang isolate abbreviations are defined in Data S1; KZ denotes Kizilsu in this study.

The smallest tested country–year cap meeting both diversity-retention criteria was *k* = 12. The resulting reduced contextual panel contained 300 sequences, including 72 from the Netherlands (24.0%), and retained 96.1% of full-tree length and 1062/1094 segregating sites (97.1%; Figure 3).

**Figure 4:**
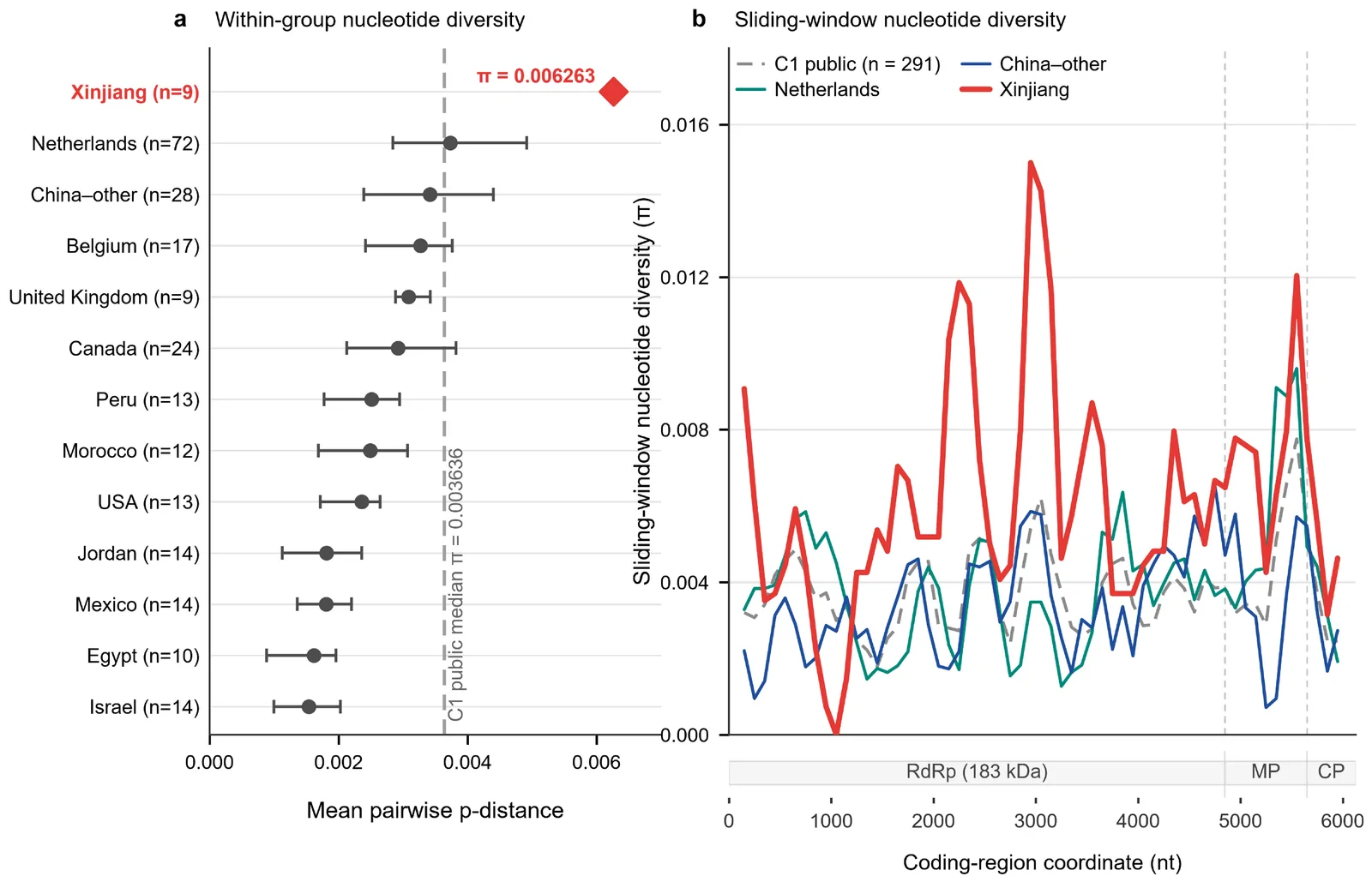
Descriptive coding-region distance and diversity summaries for ToBRFV sequence groups. (**a**) Within-group mean pairwise nucleotide distances. The sampled Xinjiang value uses all nine isolates. For each eligible country group in the reduced public background, points and horizontal intervals show the median and empirical 2.5th–97.5th percentiles from 1000 draws of eight sequences without replacement. Parentheses give the available group size, not the draw size. The dashed line shows the corresponding subsampling median for the reduced public background (*n* = 291, excluding the nine Xinjiang genomes). These distance-based summaries are separate from the DnaSP estimates reported in the text and do not estimate Xinjiang-wide population differentiation. (**b**) Sliding-window nucleotide diversity across the fixed 6129 nt replicase–*MP*–*CP* projection (300 nt windows, 100 nt step). Curves represent the nine sampled Xinjiang genomes, other Chinese sequences in the reduced panel (*n* = 28), Netherlands sequences in the reduced panel (*n* = 72) and the reduced public background (*n* = 291). Values are unsmoothed window estimates without confidence intervals; comparisons describe the sampled sequence groups.

In this descriptive comparison, country-level subsampling summaries were lower than the Xinjiang nine-genome mean pairwise distance. Median within-group distances ranged from 0.001538 for Israel to 0.003735 for the Netherlands; median descriptive between-group contrast values relative to the Xinjiang set ranged from 0.0738 for the other Chinese sequences to 0.2690 for Peru (Figure 4a; Figure S3). Xinjiang-to-public pairwise nucleotide identity ranged from 98.11% to 99.77%. Within the nine-genome Xinjiang set, sliding-window *π* peaked at coding-region positions 2801–3100 (*π* = 0.0150; Figure 4b).

Neutrality statistics, including Tajima’s *D* [21], were negative for both the full matrix and the reduced contextual panel (Tajima’s *D* = −2.65769 and −2.69878, respectively; both *p* < 0.001), with concordantly negative Fu’s *Fs*, Fu and Li’s *D* and *F*, and Achaz’s *Y*. These statistics characterize the assembled sequence panels; the nine Xinjiang genomes are summarized descriptively without neutrality inference.

In the 444-sequence maximum-likelihood tree and the primary reduced-panel tree, both inferred under TVM+F+I+R3, the nine Xinjiang sequences are distributed across multiple placements (Figure 3 and Figure S4). ToBRFV-XJ (Urumqi) paired with the Chinese 2020 sequence MZ004925 (SH-aLRT/UFBoot = 75.3/93). Four isolates, ToBRFV-YL (Yili), ToBRFV-TLF1 and ToBRFV-TLF2 (Turpan), and ToBRFV-KZ (Kizilsu), formed a strongly supported clade (98.8/100) nested among predominantly European publicly available sequences. The Hotan isolates ToBRFV-HT1 and ToBRFV-HT2 formed a supported pair (96.4/100). ToBRFV-KS (Kashgar) occurred in a clade with the Chinese sequences OR792460, OR795503 and PQ492146 (75.8/87). ToBRFV-AKS (Aksu) was placed near two Ningxia 2025 sequences, PZ099224 and PZ099225, with weak local support (0/69; inner Ningxia pair, 96.9/99). The publicly available Xinjiang sequence ToBRFV-XJAK (PX641106) grouped with OR500699 (95.5/99).

To test whether these placements depended on the composition of the public background, we repeated the tree analysis with 100 reduced contextual panels. The sample-level multi-placement pattern for the nine Xinjiang isolates was retained in all panels, and each principal placement pattern was retained in all panels except the ToBRFV–AKS–Ningxia association (98/100; Figure S5; Table S7). A second Chinese-background sensitivity analysis perturbed non-key exchangeable Chinese public records by country–year stratum while retaining the nine Xinjiang genomes, event-defining Chinese anchors and the non-Chinese reduced-panel background; all predefined placement events were retained in 7/7 assessable panels (Table S7). A complementary Xinjiang leave-one-out summary showed that each eight-genome subset retained *h* = 8 and *π* values close to the nine-genome estimate (Figure S6). PCoA likewise distributed the Xinjiang genomes across the ordination; the first two axes accounted for 25.0% and 10.0%, respectively, of the sum of positive eigenvalues (Figure S7). Root-to-tip regressions showed limited temporal structure across the five dated-tip filtering strategies (*R*^2^ = 0.155–0.193; Table S8). These analyses evaluate sensitivity of the observed placements and diversity summaries within the curated dataset, not representativeness of Xinjiang ToBRFV overall.

### 3.3. Coding-Region Variation, Sequence Quality Control and Feature Annotation of Xinjiang ToBRFV Isolates

The gap-free Xinjiang coding-region alignment allowed coding-region variants and isolate-level ORF integrity to be examined directly.

**Figure 5:**
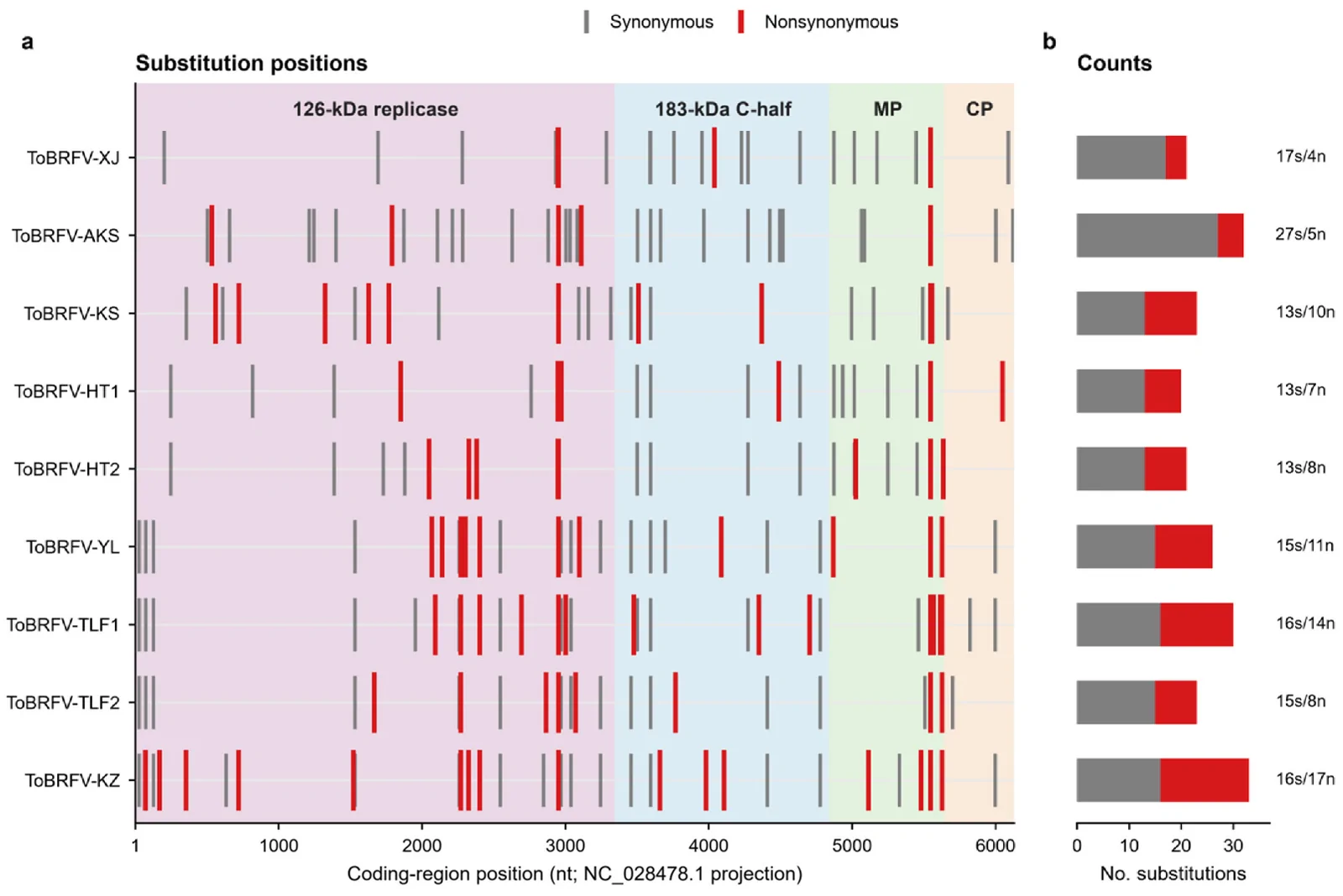
Coding-region variation in nine Xinjiang ToBRFV isolates. (**a**) Distribution of synonymous (grey) and nonsynonymous (red) substitutions relative to the external reference genome NC_028478.1 across the fixed 6129 nt coding-region projection. Each row represents one isolate, and vertical marks indicate substitution positions. Background shading identifies the 126 kDa replicase, 183 kDa C-terminal region, MP and CP coding regions. (**b**) Total synonymous (grey) and nonsynonymous (red) substitution counts for each isolate, shown as stacked bars with numerical labels. Substitutions were classified using region-local codon annotations in the reference reading frame. Under this annotation rule, ToBRFV-KS replicase substitutions were classified, although the isolate was excluded from the replicase protein and codon datasets because of the W441* premature stop (Figure S8). Xinjiang isolate abbreviations are defined in Data S1; KZ denotes Kizilsu in this study.

Relative to the reference genome NC_028478.1, each Xinjiang isolate carried 20–33 coding-region substitutions, comprising 13–27 synonymous and 4–17 nonsynonymous changes per genome. The largest number of substitutions was observed in ToBRFV-KZ (33 substitutions, including 17 nonsynonymous changes), followed by ToBRFV-AKS (32 substitutions) and ToBRFV-TLF1 (30 substitutions). At the distinct-site level, non-reference variants were distributed across the 126 kDa replicase region, the 183 kDa C-terminal region, MP and CP, with 78, 30, 26 and 8 affected positions, respectively (Figure 5). Of these Xinjiang non-reference allele states, 93 were not observed in the curated publicly available panel and 49 were shared with at least one publicly available sequence.

All nine Xinjiang MP and CP coding regions were translated without ORF-disrupting events. ToBRFV-KS contained a consensus W441* premature stop within the N-terminal coding region shared by the 126 kDa replicase and the 183 kDa readthrough ORF (Figure S8). It was therefore retained for nucleotide comparisons but excluded from protein- and codon-based analyses of both replicase regions. Thus, the Xinjiang component comprised nine sequences for nucleotide, MP and CP analyses and eight for translation-dependent analyses of each replicase region.

Next, we annotated selected sequence features in the same 444-sequence matrix to place the Xinjiang coding-region variants in a broader genomic context.

Six MP residues previously highlighted in studies of ToBRFV interactions with *Tm-2^2^*-mediated resistance were annotated in the 444-sequence matrix. All nine Xinjiang isolates carried H67/N125/K129/A134/I147/I168, the residue combination present in 437/444 sequences, whereas seven non-Xinjiang publicly available sequences carried A134T (Figure S9a).

The 126 kDa readthrough stop codon (TAG; coding-projection positions 3349–3351) was conserved in all 444 sequences; the dominant downstream 8 nt motif CAATTACA (positions 3352–3359) was present in 438/444 sequences, while six non-Xinjiang publicly available sequences carried CAATTACT. In the putative MP- and CP-proximal sequence windows defined in the reference coding projection, the MP-proximal window was more conserved than the coding-region average (variant density 0.141 versus 0.179), whereas the CP-proximal window contained substitutions observed only in the Xinjiang set, including variants unique within the curated matrix in ToBRFV-TLF1 at position 5612 and ToBRFV-HT2 at position 5635 (Figure S9b).

Dinucleotide summaries for the Xinjiang genomes were similar to the global ToBRFV profile (Figure S9c). CpG observed/expected ratios were approximately 0.82 across the coding region, 0.70 in the 183 kDa region and 0.96 in CP; UpA ratios were approximately 0.72–0.76. The maximum absolute tomato-versus-pepper difference in RSCU was 0.024.

### 3.4. Recombination Screening and Exploratory Codon-Model Analyses Across ToBRFV Coding Regions

Recombination screening found no signal that was consistently supported across methods in the 85-sequence gap-free core. Exact-type-collapsed full and reduced dataset screens gave the same broad outcome (Table S9). Isolated MaxChi signals lacked corroboration from other screens, and the RDP5 sensitivity filter retained no unique recombination events. Four-gamete incompatibilities were concentrated near the *MP*–*CP* junction (Figure S10) but did not establish a recombination breakpoint.

**Figure 6:**
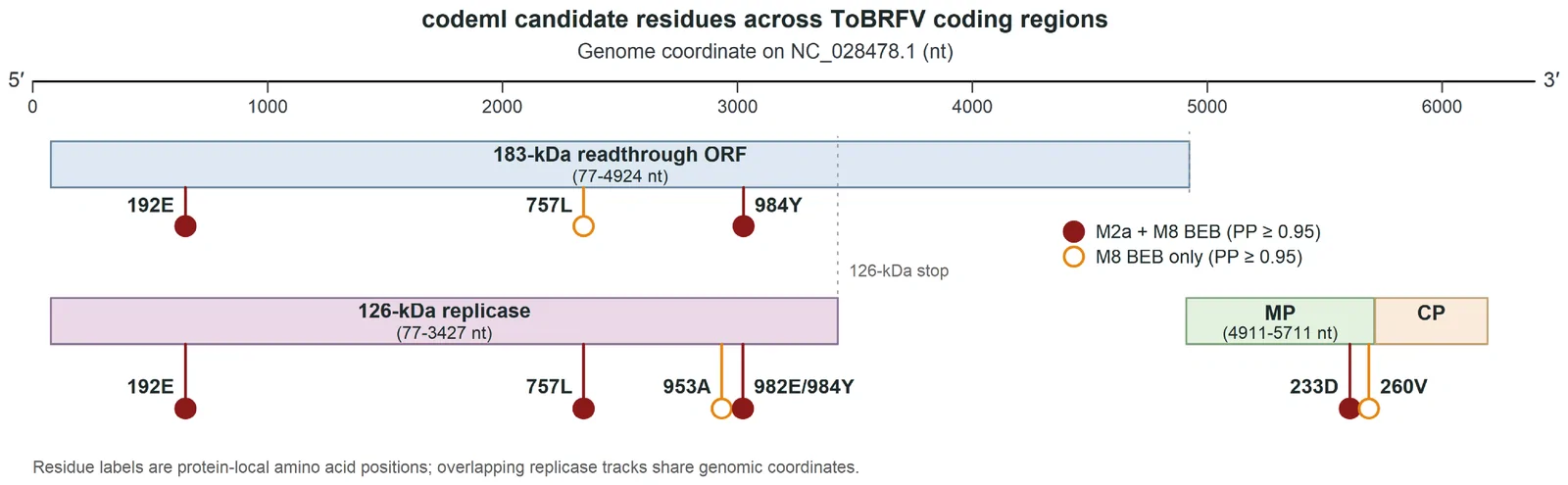
Genomic distribution of codeml candidate residues. The scale shows nucleotide coordinates on the ToBRFV reference genome NC_028478.1. Candidate residues are plotted on the corresponding protein-region tracks: 183 kDa readthrough replicase ORF, 126 kDa replicase, *MP* and *CP*. Filled dark-red circles indicate residues supported by both codeml M2a and M8 BEB summaries at posterior probability ≥ 0.95, and open orange circles indicate residues supported only by M8. The 183 kDa readthrough ORF includes the 126 kDa replicase region, so residues on the 183 kDa and 126 kDa tracks can share nucleotide coordinates but refer to protein-local amino acid numbering within each analyzed region. *CP* showed no BEB candidate residues. The plotted sites are sequence-based screening candidates and are not experimentally validated adaptive or functional residues. The 126 kDa and 183 kDa replicase candidates were not subjected to final duplicate-retaining sensitivity comparisons, and MP candidate support was not fully concordant in the duplicate-retaining FEL sensitivity analysis. Full likelihood-ratio tests, BEB probabilities, FEL sensitivity results, convergence notes, exclusions and input counts are provided in Table S10. Xinjiang isolate abbreviations are defined in Data S1; KZ denotes Kizilsu in this study.

In the exact-type-collapsed codeml analyses, the M0 estimates of ω were 0.219 for the 126 kDa replicase region and 0.171 for the full 183 kDa readthrough coding region. Both M1a–M2a and M7–M8 comparisons favoured models containing an *ω* > 1 site class in each region (Table S10). Across M2a and M8, BEB identified five and three candidate sites, respectively, at posterior probabilities ≥ 0.95; all three 183 kDa candidates were also identified in the shared N-terminal region of the 126 kDa analysis (Figure 6). The 183 kDa M8 estimates remained provisional because numerical stability across alternative starting values had not been verified. Replicase candidate-site inferences were exploratory, with no completed duplicate-retaining sensitivity comparisons.

*CP* showed no site-model support for positive selection under either duplicate treatment, with M0 estimates of *ω* of 0.184 and 0.155 for collapsed and duplicate-retaining inputs, respectively. For *MP*, both collapsed codeml comparisons supported models allowing *ω* > 1; BEB identified MP-233D under both models and MP-260V under M8 only. In the duplicate-retaining FEL analysis, neither site reached *p* < 0.05: MP-233 showed directionally consistent but borderline evidence (*p* = 0.053), whereas MP-260 was not supported (*p* = 0.357). Because this comparison changed both duplicate handling and statistical framework, the *MP* candidates remained provisional. Full model and sensitivity results are provided in Table S10.

## 4. Discussion

This study links the multi-virus composition of symptomatic protected-tomato plants in Xinjiang with coding-region variation among nine local ToBRFV genomes. It extends previous Chinese ToBRFV reports and regional tomato-virus surveys [5,9,10,11,12] by integrating pooled sRNA-seq-based virus discovery, individual RT-PCR/Sanger confirmation and comparative genome analysis within the same field collection. ToBRFV was the most frequently detected of the nine viruses, but most sampled plants contained multiple viruses, and the nine sequenced ToBRFV genomes occupied several phylogenetic positions. Together, these observations show that frequent ToBRFV detection in symptomatic plants coexisted with diverse virus combinations and sequence heterogeneity among the sampled ToBRFV isolates.

The high detection proportion of ToBRFV occurred within a heterogeneous multi-virus context. The 116 symptomatic plants contained 47 distinct virus-combination profiles, with frequent co-detection of ToBRFV with TMV and TYLCV. Consequently, a positive ToBRFV result alone would not characterize the full set of viruses detected in an individual plant. Mixed infections can alter virus accumulation and symptom expression in plant-virus systems [3], and experimental work on ToBRFV-PepMV mixed infections demonstrated increased PepMV accumulation and altered symptom severity [4], providing a biological rationale for considering mixed infections during diagnosis. However, the common pairings observed here do not establish preferential associations or synergistic interactions, particularly given the high overall detection proportion of ToBRFV. Their immediate diagnostic significance is the need for multi-virus testing rather than attributing overlapping symptoms to the most frequently detected virus alone.

Xinjiang is a relevant surveillance setting because protected vegetable production is important to the region’s vegetable supply [1], and previous work has documented tomato and vegetable viruses in Xinjiang and southern Xinjiang [2,10,12]. For a mechanically transmissible and highly persistent tobamovirus such as ToBRFV, intensive protected cultivation and the movement of plant materials through production chains are reasonable surveillance concerns. The present dataset addresses this setting at the level of symptomatic-plant detection and coding-sequence context; it does not identify introduction routes, sources or regional frequency.

The nine Xinjiang genomes retained multiple phylogenetic placements across alternative public-sequence backgrounds. This sensitivity analysis was particularly relevant because records assigned to the Netherlands in the source metadata accounted for nearly half of the public dataset, and geographic sampling bias can affect reconstructions of viral locations and movements [22]. ToBRFV-specific Nextstrain analyses of outbreaks in the Netherlands and a later expanded global build illustrate how genomic data are most informative when interpreted together with epidemiological, production-chain or movement metadata [23,24]. A recent Portuguese study provides another example of placing local ToBRFV genomes in a global phylogenetic context [25]. In the present dataset, close phylogenetic relationships alone were not treated as evidence of an introduction route or direction. The additional Chinese-background analysis gave concordant results while retaining predefined reference sequences, supporting stability under the specific background changes tested. Nevertheless, placement recurrence should be distinguished from branch support: the ToBRFV-AKS association with the Ningxia sequences was repeatedly recovered but remained weakly supported locally. Together, these analyses support heterogeneity among the sampled genomes without recovering diversity absent from the public database. Establishing introduction numbers, sources or transmission direction would require denser sampling linked to seed-lot and upstream production-chain records.

The 444-sequence matrix contained 344 exact coding-sequence types, representing strict sequence identity classes, despite low overall nucleotide diversity (π = 0.00354). Global ToBRFV comparisons and population-structure analyses describe low nucleotide diversity or strong purifying constraint in ToBRFV datasets [13,26], while a genus-wide tobamovirus survey found low genomic variation and prevalent purifying selection across related tobamovirus species, including ToBRFV [27]. Canadian greenhouse data further show that local production systems can contain closely related but distinguishable ToBRFV genomes [28]. Because exact coding-sequence types can differ by a single nucleotide, their number should not be equated with the number of deeply divergent lineages. All nine Xinjiang genomes were distinct and unmatched in the curated public background, with 139 biallelic segregating sites and π = 0.00626. Their higher descriptive diversity relative to the sampled public groups, together with their distribution across the phylogeny and PCoA, indicates heterogeneity within the selected genome set rather than a regional estimate of diversity or geographic exclusivity. The negative Tajima’s D values in the full and reduced matrices are consistent with an excess of low-frequency variants relative to the standard neutral expectation [21]. However, these geographically and temporally heterogeneous sequence collections do not distinguish demographic expansion from selective or sampling-related effects [29], so the neutrality results were not treated as independent evidence of recent population expansion.

Recombination screening yielded no signal that was consistently supported across methods in the analyzed ToBRFV panels. PHI, NSS and sliding-window analyses did not corroborate the isolated MaxChi signals, and expanded screening of the exact-type-collapsed full and reduced matrices gave the same broad outcome. This agrees with an earlier ToBRFV analysis that reported no recombination evidence using the Genetic Algorithm for Recombination Detection (GARD) method [30]. These findings remain screening-level observations rather than proof of a strictly non-recombinant history: no specific breakpoints or parental sequences were inferred, and undetected recombination cannot be excluded.

In codon-based analyses of the reduced contextual panel, *CP* showed no significant support for models containing a site class with *ω* > 1 in either the exact-type-collapsed or duplicate-retaining codeml analyses; the corresponding M0 estimates of *ω* were 0.184 and 0.155. This pattern is consistent with overall purifying constraint and previous ToBRFV comparisons [13,26]. Site-level inferences for *MP* were more sensitive to analytical treatment. Earlier sequence analyses have also reported candidate selection signals in *MP* [30]. The collapsed codeml analysis identified MP-233 and MP-260 as candidates, whereas duplicate-retaining FEL analysis provided borderline, directionally consistent evidence for MP-233 and no significant support for MP-260. Differences in model assumptions and site-support criteria may contribute to this discrepancy [31], but the effects of statistical framework and duplicate handling could not be separated because both changed together. These residues therefore remain model-dependent, sequence-based candidates.

Both the 126 kDa and 183 kDa readthrough replicase analyses favoured models containing a site class with *ω* > 1. For the 183 kDa region, this support coexisted with an M0 estimate of ω of 0.171, illustrating that overall purifying constraint does not preclude model-based evidence for a subset of sites with *ω* > 1. The reported 183 kDa BEB candidates were located within the N-terminal coding region shared with the 126 kDa replicase and were also identified in the 126 kDa analysis. Because these analyses include overlapping sequence data, their agreement does not constitute independent statistical replication. Neither replicase analysis was evaluated in a final duplicate-retaining sensitivity comparison, so the candidate-site inferences shown in Figure 6 remain exploratory. Together, these findings distinguish a *CP* constraint pattern that was consistent across the tested treatments from more provisional site-level signals in MP and the replicase. Independent sequence datasets and additional model-based analyses are needed to assess the robustness of these signals, while their biological significance requires functional evaluation.

Complete coding-region coverage did not always coincide with predicted coding integrity. All nine Xinjiang genomes had intact MP and CP coding regions, whereas the ToBRFV-KS consensus sequence contained a W441* premature stop within the 126 kDa replicase region (Figure S8), distinct from the normal readthrough stop. ToBRFV-KS was therefore retained for nucleotide comparisons but excluded from protein- and codon-based analyses of both the 126 kDa replicase and the 183 kDa readthrough ORF. The biological significance of W441* remains unresolved.

Local nucleotide variation coexisted with conservation of several surveyed sequence features. All nine Xinjiang genomes matched the predominant public-matrix residue combination in MP, a protein that is experimentally implicated in host-resistance interactions [7]. The annotated replicase readthrough stop codon was conserved across the 444-sequence matrix, whereas limited variation occurred in the downstream motif. Among the reference-defined windows upstream of the MP and CP start codons, the MP-upstream window had a lower variant density than the coding-region average, whereas the CP-upstream window contained variants observed only in the Xinjiang set within this matrix. These windows were defined by reference coordinates, with experimental promoter mapping in TMV providing biological context for their examination [19]. CpG and UpA profiles were similar between the Xinjiang genomes and the public sequences. A separate comparison based on recorded host metadata showed a maximum absolute tomato-versus-pepper RSCU difference of 0.024. Previous ToBRFV and tobamovirus-wide analyses reported gene-specific codon-usage or genome-wide selection patterns [27,30]. Together, these observations place local sequence variation within the broader genomic context of ToBRFV. The effects of these sequence features on gene expression or viral phenotype were not measured in this study.

The apparent discrepancy between pooled sRNA-seq and individual RT-PCR/Sanger detections should be interpreted as a limitation of the discovery workflow rather than as a direct conflict in prevalence estimates. The sRNA-seq results were generated from 17 geographically defined composite libraries, whereas RT-PCR/Sanger confirmation was performed on 116 individual symptomatic plants. Therefore, the pool-level sRNA percentages do not estimate individual-plant prevalence and cannot be used to compare assay sensitivity directly. However, this difference in sampling unit alone does not fully explain the large directional contrast observed for ToBRFV and TMV. Similar studies comparing pooled HTS with individual plant-level PCR have shown that discordant detections can reflect low viral abundance, incomplete genome coverage and predefined reporting thresholds, and multi-laboratory evaluations of sRNA pipelines have further shown that sequencing depth, bioinformatic workflow, reference-database completeness and cutoff settings can affect virus recovery [32,33]. In the present study, pooled sRNA-seq was therefore used primarily as a virus-discovery screen, whereas individual RT-PCR/Sanger results were used as the basis for plant-level detection profiles. The available summary outputs do not allow every sRNA-negative but RT-PCR-positive pool to be resolved mechanistically, so the low pool-level recovery of ToBRFV should not be interpreted as evidence of low field occurrence.

Root-to-tip regressions showed limited clock-like structure across the five dated-tip filtering strategies (R2 = 0.155–0.193). These regressions are exploratory and do not replace formal evaluation of temporal signal [34,35]. As no formal temporal-signal evaluation was performed, the trees were used to compare sequence relationships rather than date introductions or reconstruct transmission chronology.

These findings document complex multi-virus co-detection in symptomatic protected-tomato samples from Xinjiang and sequence heterogeneity among nine local ToBRFV genomes. The combined detection and sequence data provide a local genomic resource for ToBRFV surveillance and comparative analysis and place Xinjiang coding-region genomes within the current global sequence background. Because sampling targeted symptomatic plants and the genome set was small, these observations cannot establish regional prevalence, population-wide ToBRFV diversity or transmission history. Representative sampling and broader genome coverage are needed to assess regional virus occurrence and sequence diversity, while reliable sampling dates, seedling-source information and production-chain records are needed to investigate epidemiological relationships.

## Data Availability

Raw small-RNA sequencing data have been deposited in the China National Center for Bioinformation (CNCB) under BioProject PRJCA074566.

## Supplementary Materials

The following supporting information can be downloaded at https://www.mdpi.com/article/10.3390/v18101127/s1. The supporting information comprises Figures S1–S10, Tables S1–S10, Methods S1–S3 and Data S1–S3. Figure S1: Pool-level viral-reference read fractions and clean-read small-RNA size summaries in the 17 composite sRNA-seq libraries. Figure S2: Minimum-spanning network of exact aligned ToBRFV coding-sequence types. Figure S3: Descriptive between-group pairwise p-distance contrasts between the nine Xinjiang ToBRFV genomes and public country groups. Figure S4: Reduced-panel maximum-likelihood phylogram showing the phylogenetic placement of Xinjiang ToBRFV genomes with branch lengths drawn to scale. Figure S5: Stability of the principal Xinjiang phylogenetic placements across 100 replicate reduced contextual panels. Figure S6: Sample-level Xinjiang leave-one-out diversity summary for the nine ToBRFV genomes. Figure S7: Principal-coordinate analysis of the 444-sequence nucleotide p-distance matrix. Figure S8: Sanger trace assessment of the ToBRFV-KS p126 W441* site. Figure S9: Sequence-based annotation of selected features in the curated 444-sequence ToBRFV coding-region matrix. Figure S10: Four-gamete/MaxChi recombination sensitivity screen. Table S1: 17 sRNA-seq composite pools and viral contig counts. Table S2: RT-PCR confirmation primers. Table S3: ToBRFV complete-genome amplification primers. Table S4: pool-level sRNA-seq detections and individual-level RT-PCR/Sanger confirmations. Table S5: ten most frequent virus-combination profiles and pairwise virus co-detection counts among 116 symptomatic tomato samples. Table S6: RT-PCR summary by sampling origin. Table S7: placement-stability summaries for replicate reduced contextual panels and Chinese-background sensitivity perturbations. Table S8: temporal-signal diagnostics. Table S9: recombination sensitivity screening of exact coding-sequence types in the full 444-sequence dataset and the primary 300-sequence reduced contextual panel. Table S10: selection-model audit and candidate-site sensitivity summary. Methods S1: Coding-region alignment and dataset construction. Methods S2: Phylogenetic analyses and genetic diversity. Methods S3: Codon models and sensitivity analyses. Data S1: Accession-level curation of ToBRFV sequences, including sample metadata, sequence-quality metrics, inclusion and exclusion status, and exact coding-sequence duplicate status. Data S2: Clade and marker cross-reference for 444 ToBRFV sequences, including rule-based clade assignments, reference-tree clade labels, marker support and nearest public-sequence matches. Data S3: Individual RT-PCR/Sanger detection matrix for nine viruses in 116 symptomatic tomato samples, including sampling origins, pooled-library identifiers, virus counts and co-detection profiles. Reference [36] is cited in Supplementary Table S2.
