# Supplementary material for: Virome Profiling via sRNA-Seq and Sample-Level Coding-Region Diversity of Tomato Brown Rugose Fruit Virus in Protected Tomato Crops in Xinjiang
Source: Viruses. 2026 Oct 11;18(10):1127. doi: 10.3390/v18101127 (PMC13709767; doi:10.3390/v18101127)
Supplement: Supplementary file 1 [file viruses-18-01127-s001.zip › Supplementary_Figures_S1-S10_Viruses_20260919.pdf]

viruses

MDPI

Supplementary Materials

Supplementary Figures S1–S10

Virome Profiling via sRNA-Seq and Sample-Level Coding-Region Diversity of Tomato brown rugose fruit virus in Protected Tomato Crops of Xinjiang

Figures accompanying the manuscript. Methods S1–S3, Tables S1–S10 and Data S1–S3 are provided as separate files.

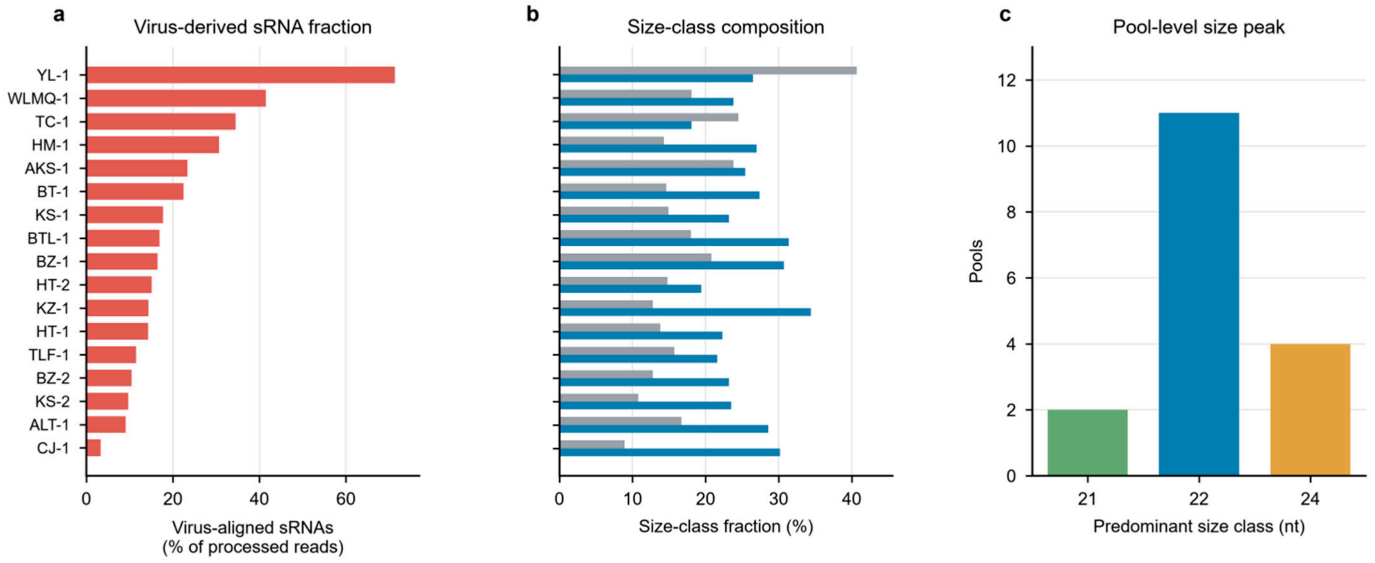

Figure S1. Pool-level viral-reference read fractions and clean-read small-RNA size summaries in the 17 composite sRNA-seq libraries. (a) Percentage of processed reads aligned to viral references in each composite pool. Pools are ordered by decreasing viral-reference read fraction. (b) Cutadapt-derived percentages of 21-nt (grey) and 22-nt (blue) small RNAs in the same pool order. (c) Number of pools in which each clean-read size class was the predominant peak. Pool-level cutadapt length-distribution files provided the size summaries; viral-reference mapping logs provided panel a.

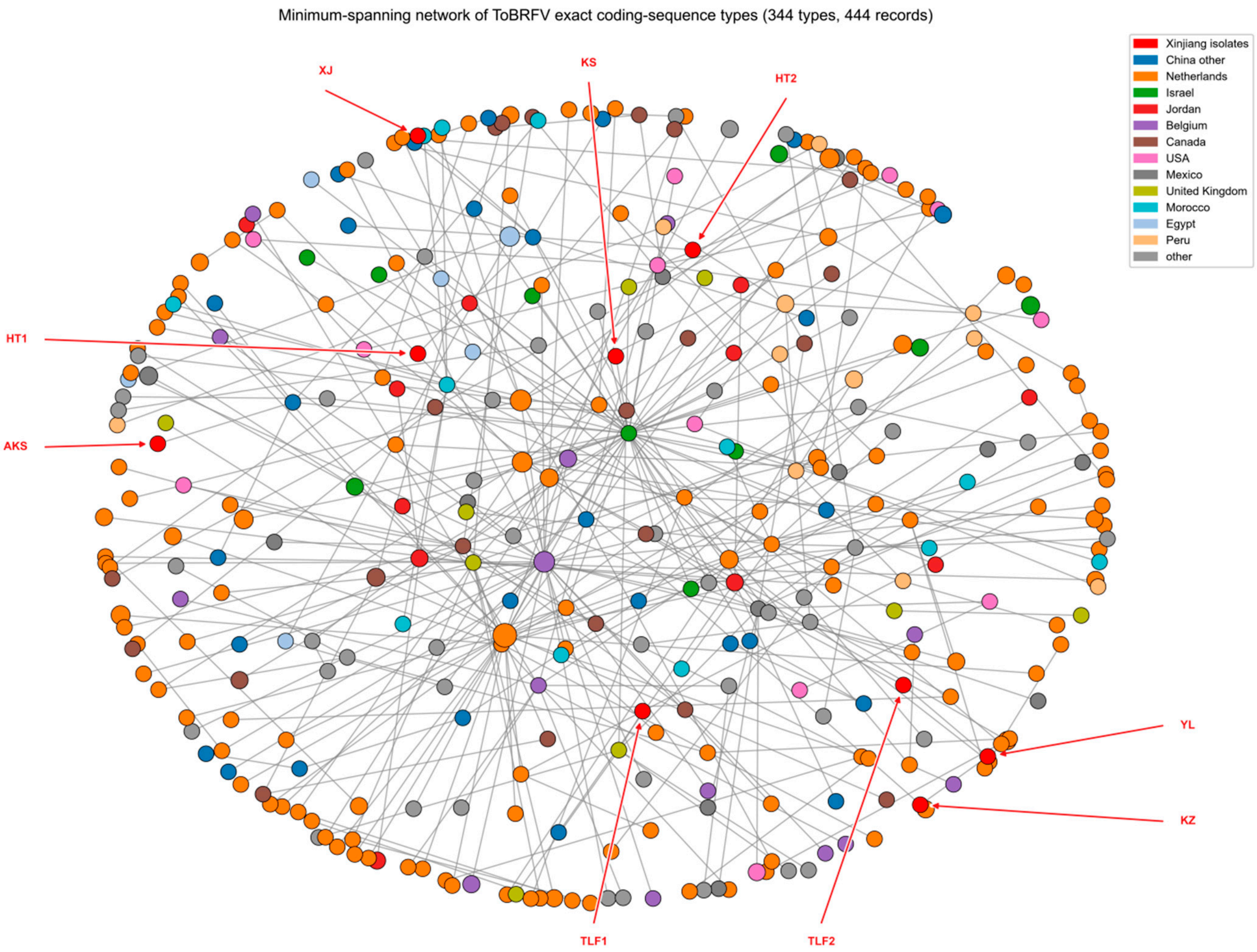

Figure S2. Minimum-spanning network of exact aligned ToBRFV coding-sequence types. Node size indicates the number of records per sequence type; the nine Xinjiang genomes are highlighted and each was unique within the curated dataset. Xinjiang isolate abbreviations are defined in Supplementary Data S1; KZ denotes Kizilsu in this study.

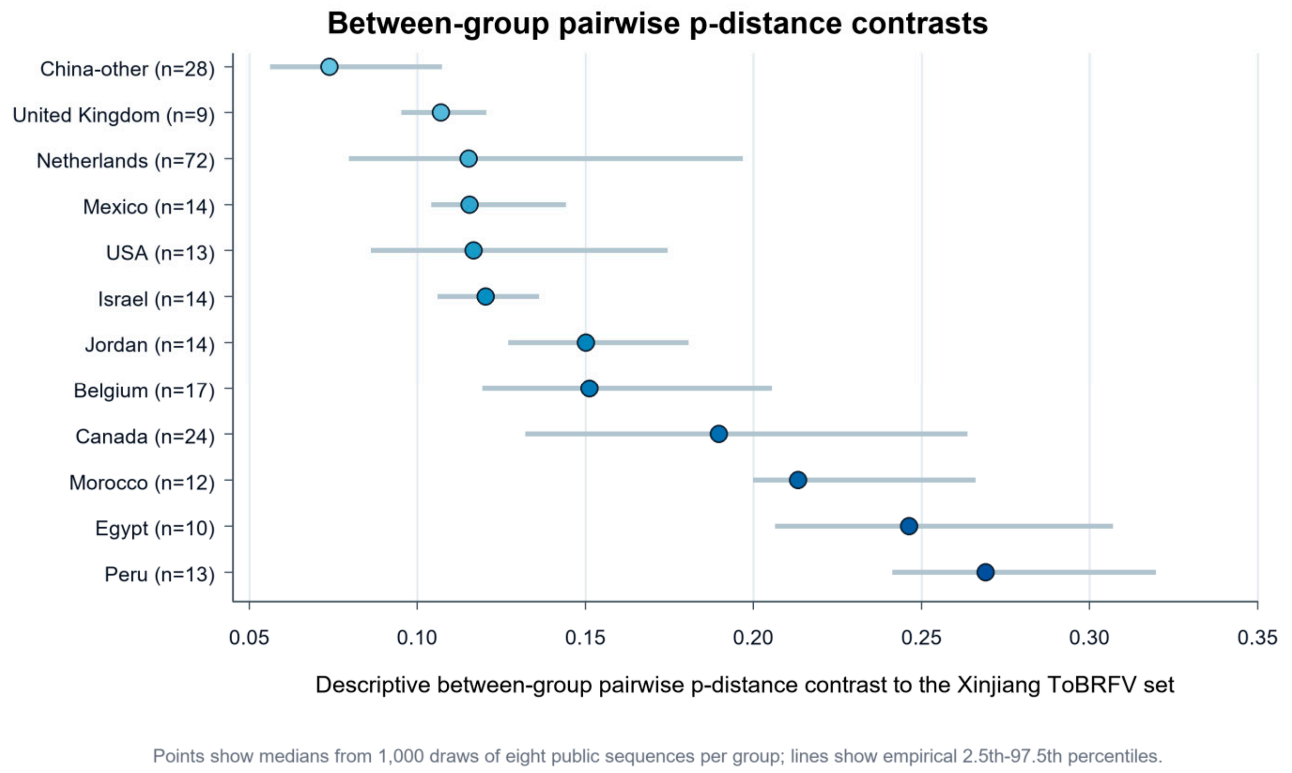

Figure S3. Descriptive between-group pairwise p-distance contrasts between the nine Xinjiang ToBRFV genomes and public country groups. Points and horizontal lines show medians and empirical 2.5th-97.5th percentiles from 1,000 draws of eight public sequences per group (eligible group size >=8). All nine Xinjiang genomes were retained in each comparison. Values describe these sequence groups; representative population differentiation or transmission-history inference requires representative sampling and epidemiological data.

Figure S4. Reduced-panel ToBRFV maximum-likelihood phylogram

Page 1/5; tips 1-60 of 300

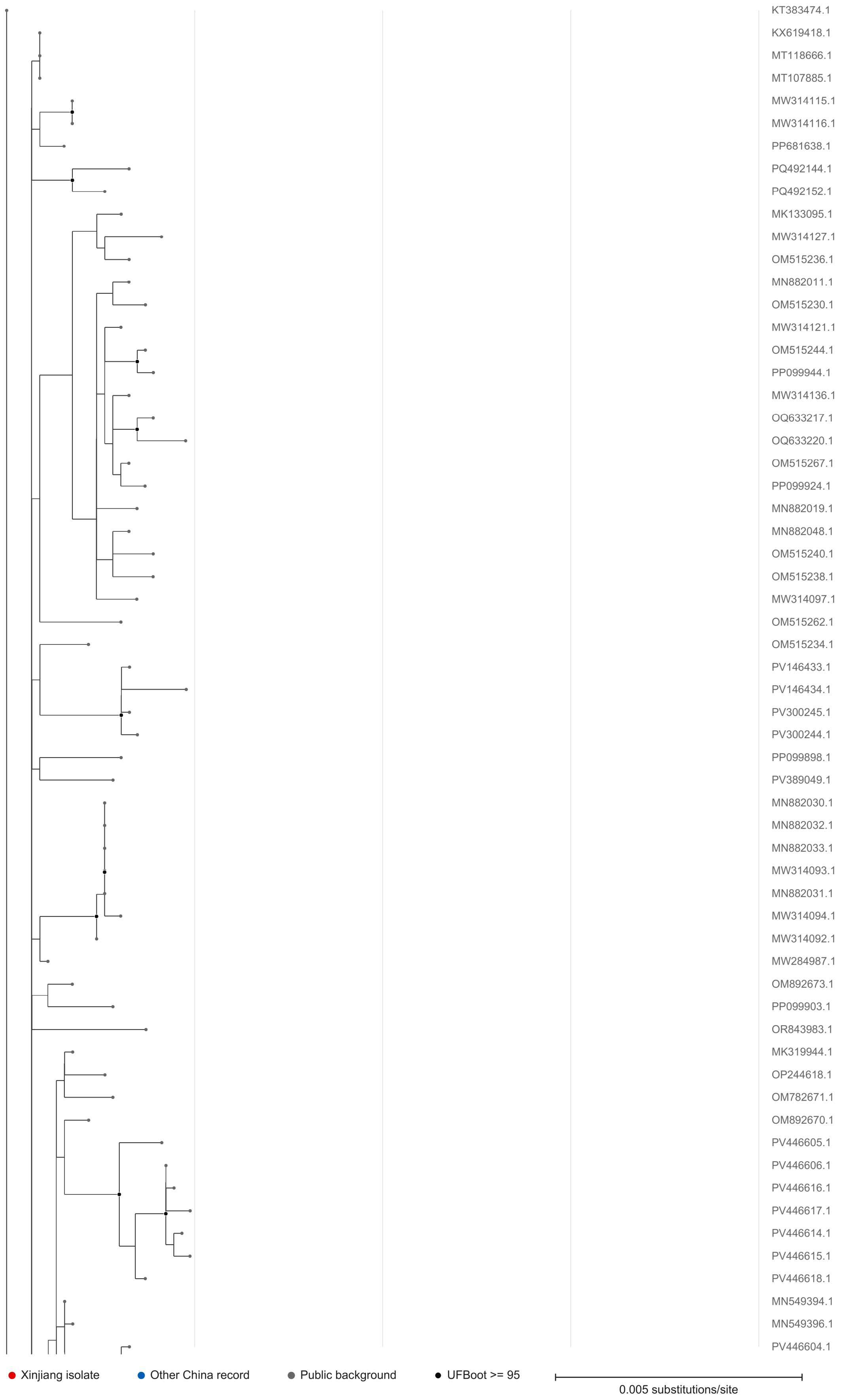

Figure S4. Reduced-panel ToBRFV maximum-likelihood phylogram

Page 2/5; tips 61-120 of 300

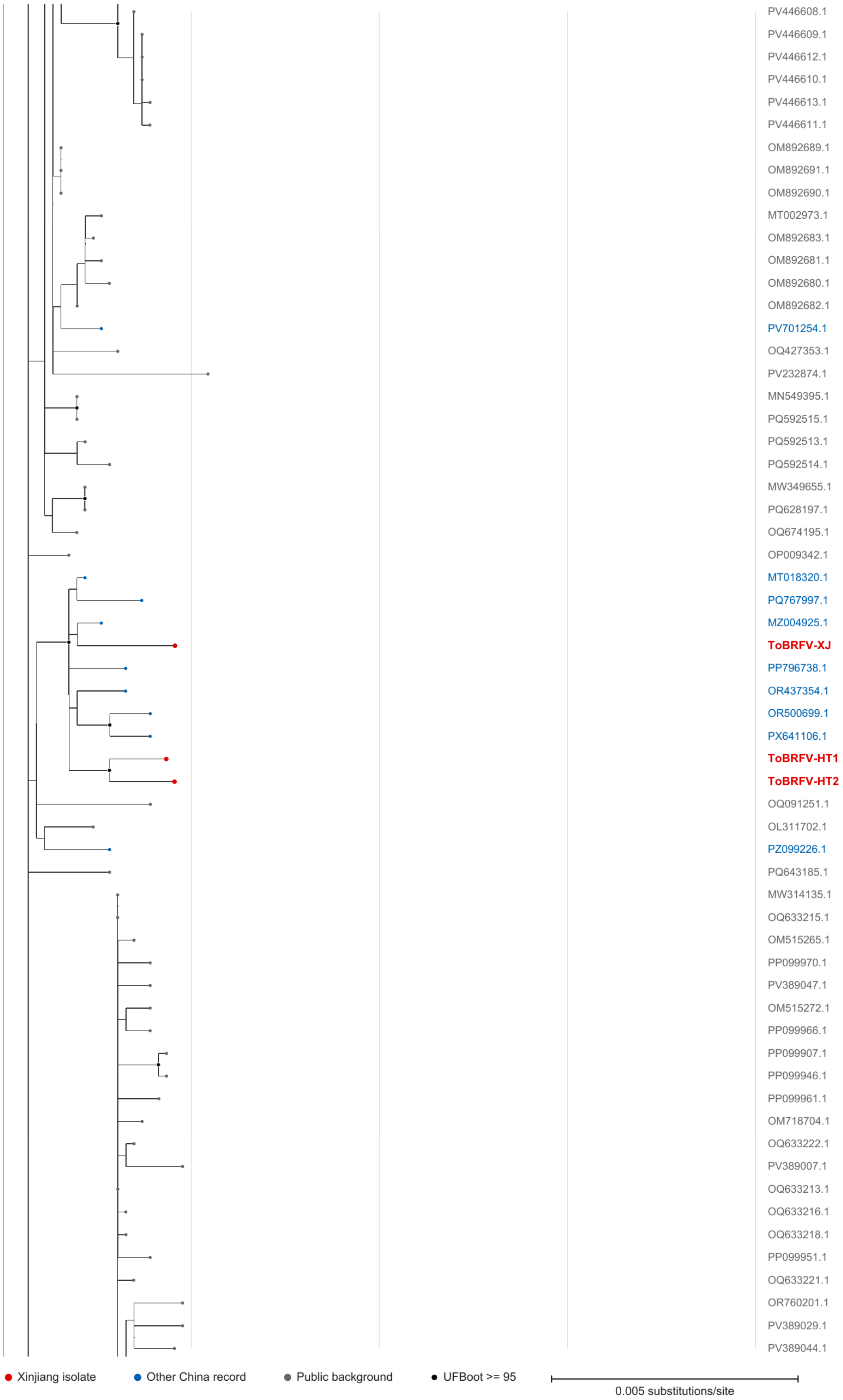

Figure S4. Reduced-panel ToBRFV maximum-likelihood phylogram

Page 3/5; tips 121-180 of 300

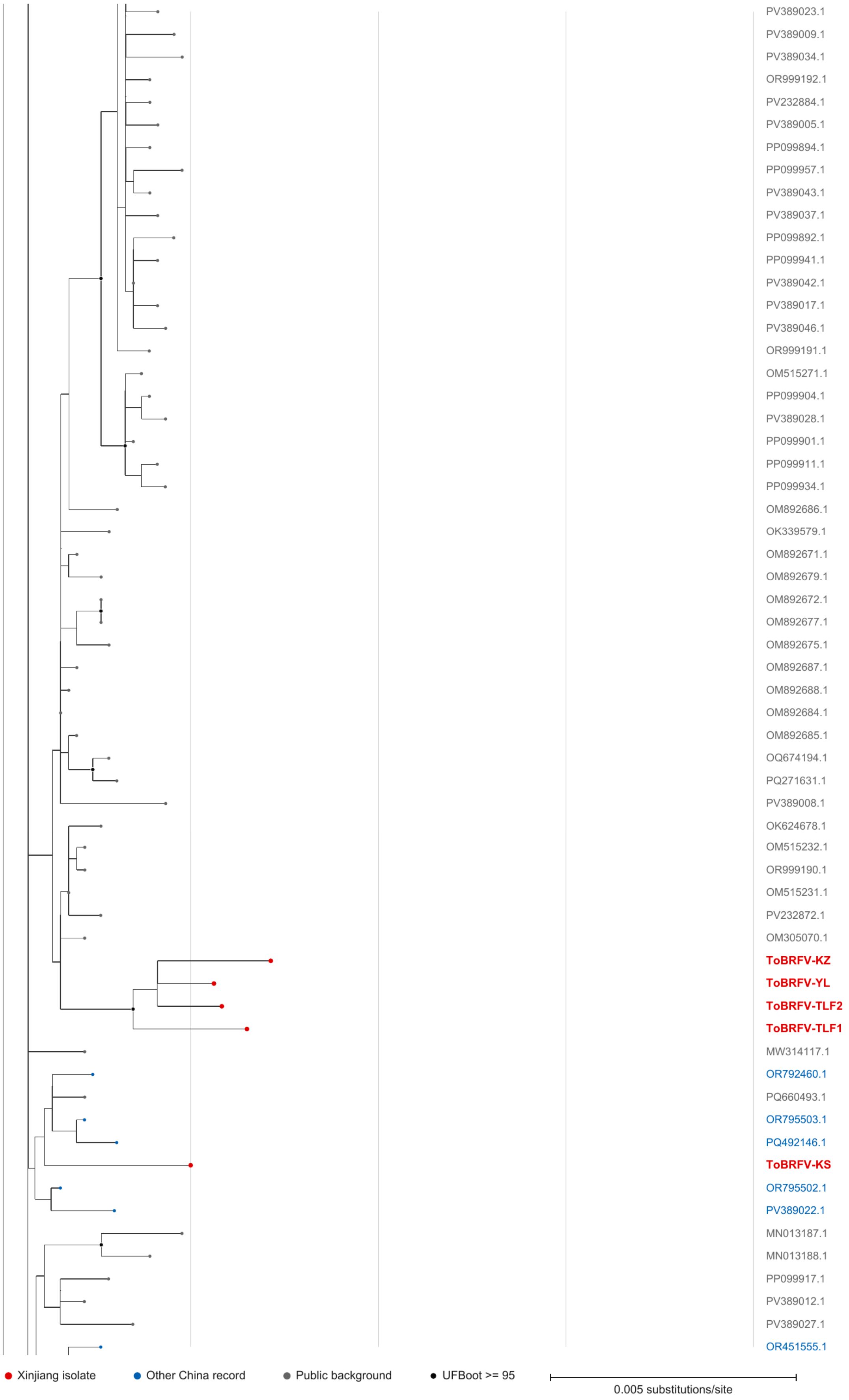

Figure S4. Reduced-panel ToBRFV maximum-likelihood phylogram

Page 4/5; tips 181-240 of 300

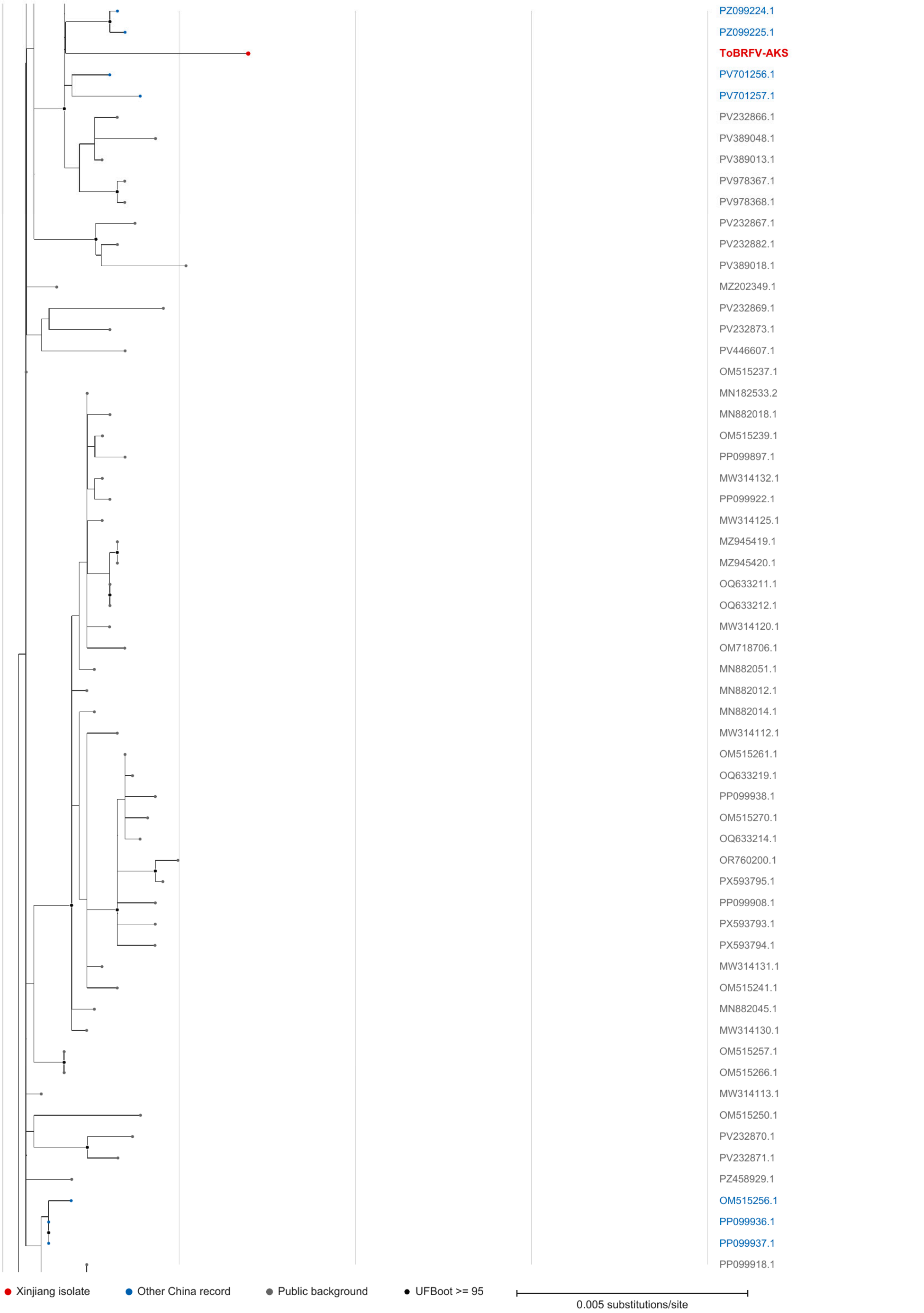

Figure S4. Reduced-panel ToBRFV maximum-likelihood phylogram

Page 5/5; tips 241-300 of 300

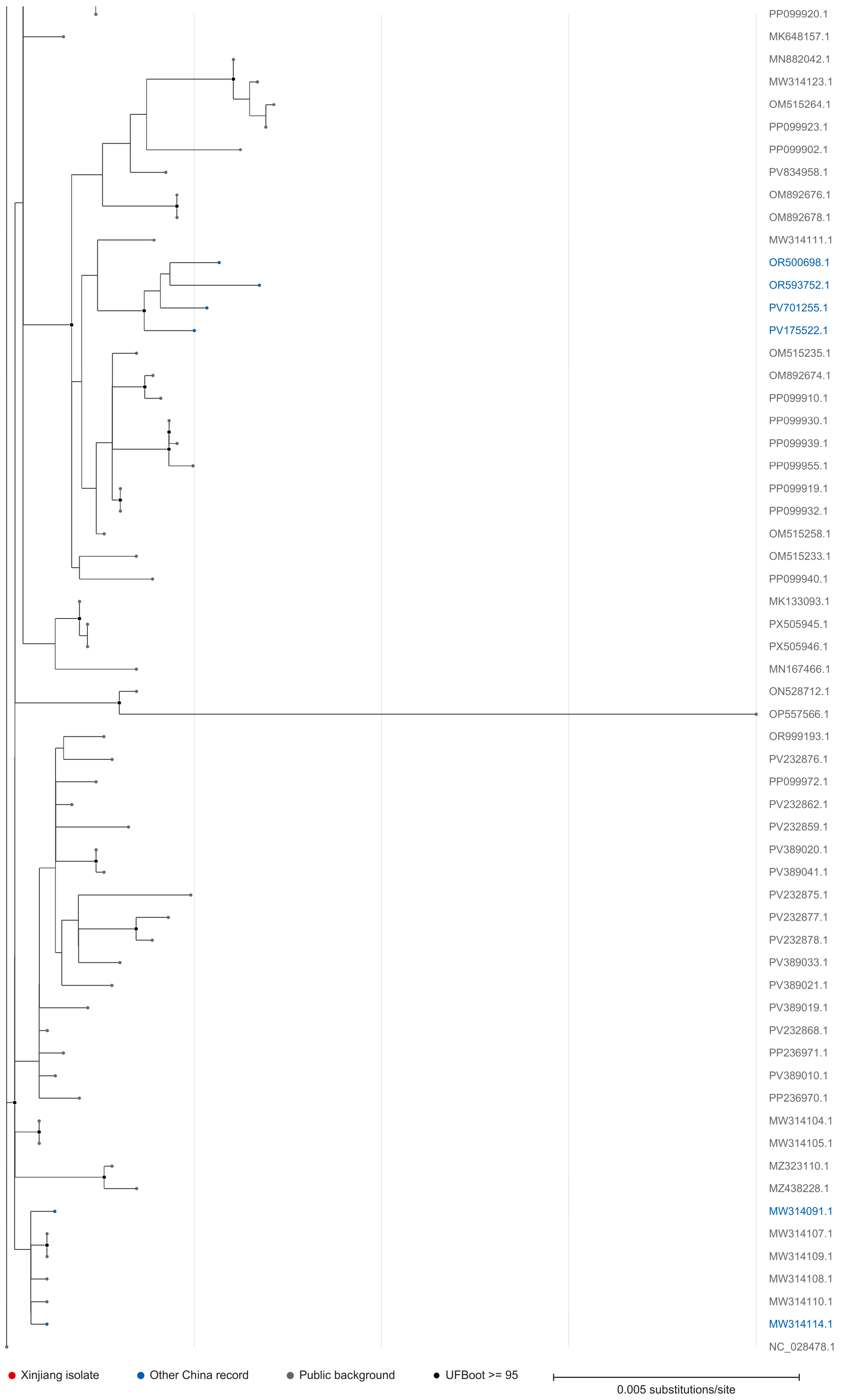

Figure S4. Reduced-panel maximum-likelihood phylogram showing the phylogenetic placement of Xinjiang ToBRFV genomes. The 300-sequence tree is displayed as a branch-length phylogram split across five pages while retaining the original tip order and a common horizontal scale. Xinjiang isolates are highlighted in red, other Chinese records in blue, and other public background records in grey. Small black nodes mark UFBoot >=95; complete SH-aLRT/UFBoot labels and page assignments are provided in Fig_S4_reduced_panel_phylogram_20260919_node_support.tsv and Fig_S4_reduced_panel_phylogram_20260919_tip_status.tsv. The scale bar indicates substitutions per site. Xinjiang isolate abbreviations are defined in Supplementary Data S1; KZ denotes Kizilsu in this study.
The underlying topology is the primary reduced contextual tree inferred from the 6,129-nt concatenated replicase-MP-CP alignment using IQ-TREE 3.0.1 under TVM+F+I+R3 with 1,000 UFBoot and 1,000 SH-aLRT replicates. The drawing preserves the Newick tip order and branch lengths; no additional pruning, rerooting or tree inference was performed for this figure.

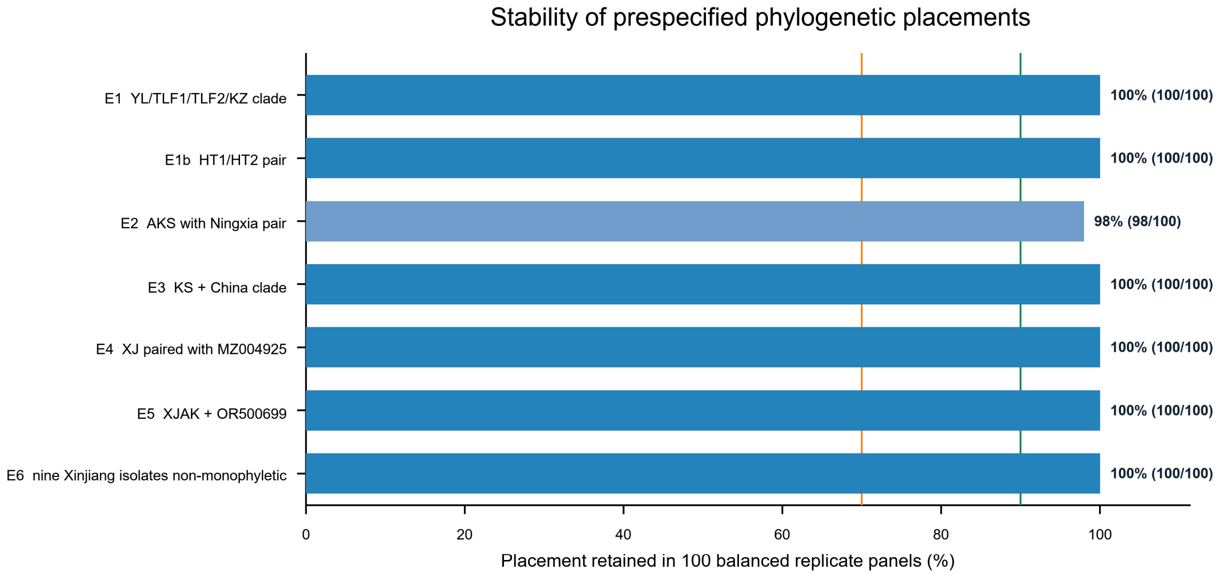

Figure S5. Stability of the principal Xinjiang phylogenetic placements across 100 replicate reduced contextual panels.

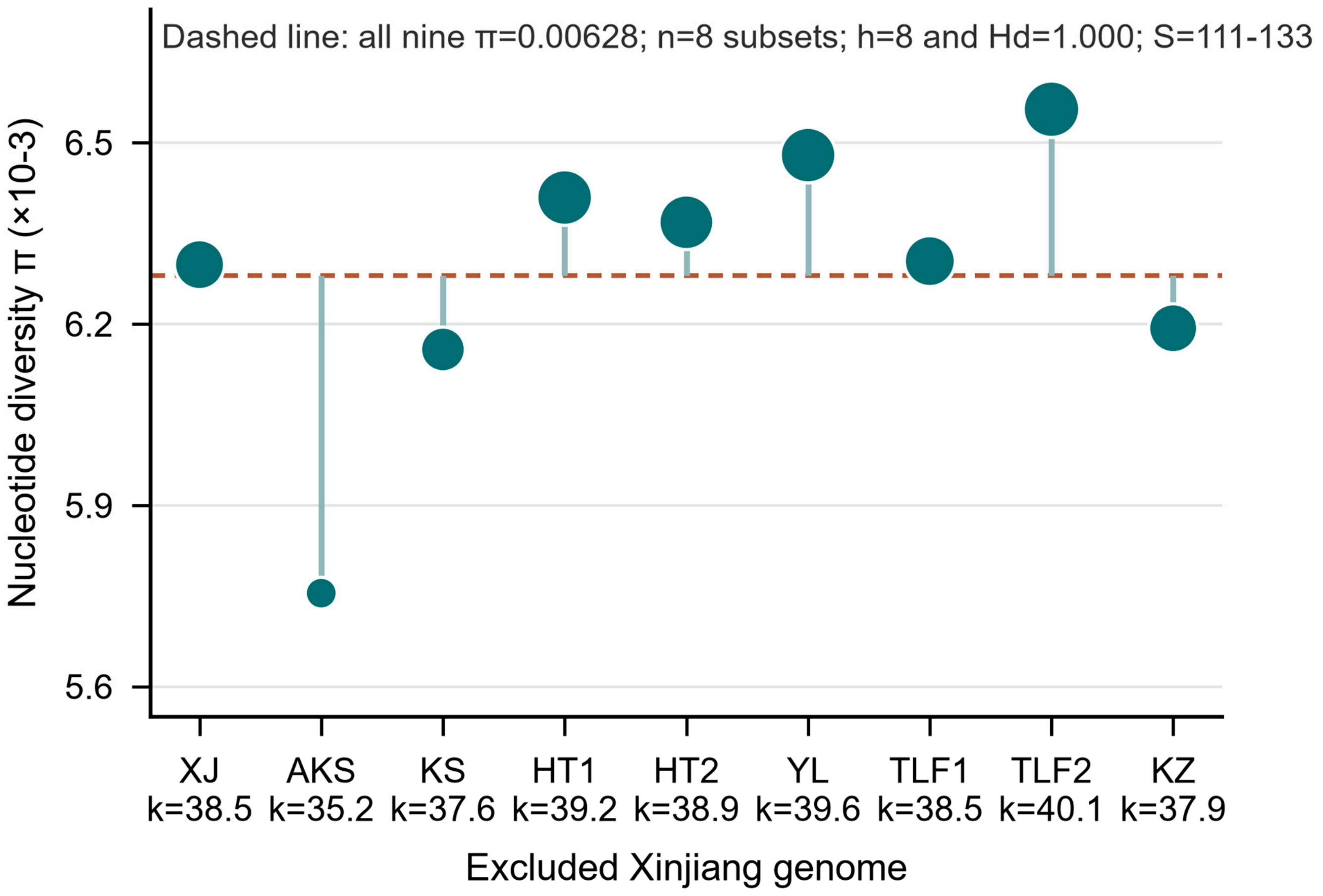

Figure S6. Sample-level Xinjiang leave-one-out diversity summary for the nine ToBRFV genomes. Each point summarizes the eight-sequence subset after excluding one Xinjiang genome. Values show haplotype count (h), average pairwise nucleotide differences (k) and nucleotide diversity (π) relative to the full nine-genome Xinjiang set; the panel is a sensitivity summary and not a regional rarefaction analysis. Xinjiang isolate abbreviations are defined in Supplementary Data S1; KZ denotes Kizilsu in this study.

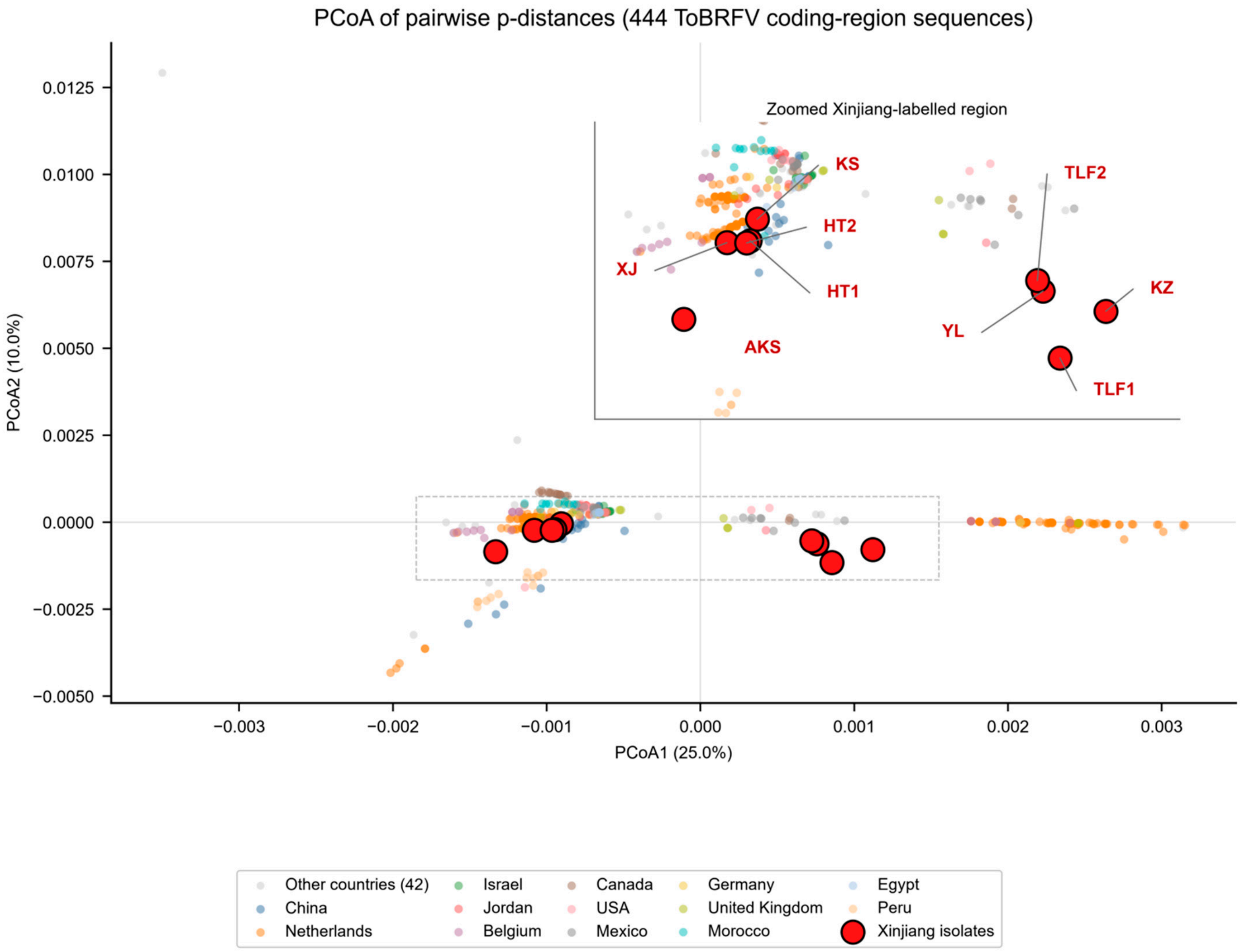

Figure S7. Principal-coordinate analysis of the 444-sequence nucleotide p-distance matrix. Xinjiang isolates are highlighted and labelled; public sequences are coloured by country. Axis 1 and axis 2 account for 25.0% and 10.0%, respectively, of the sum of positive eigenvalues. The panel shows nucleotide-distance ordination. Xinjiang isolate abbreviations are defined in Supplementary Data S1; KZ denotes Kizilsu in this study.

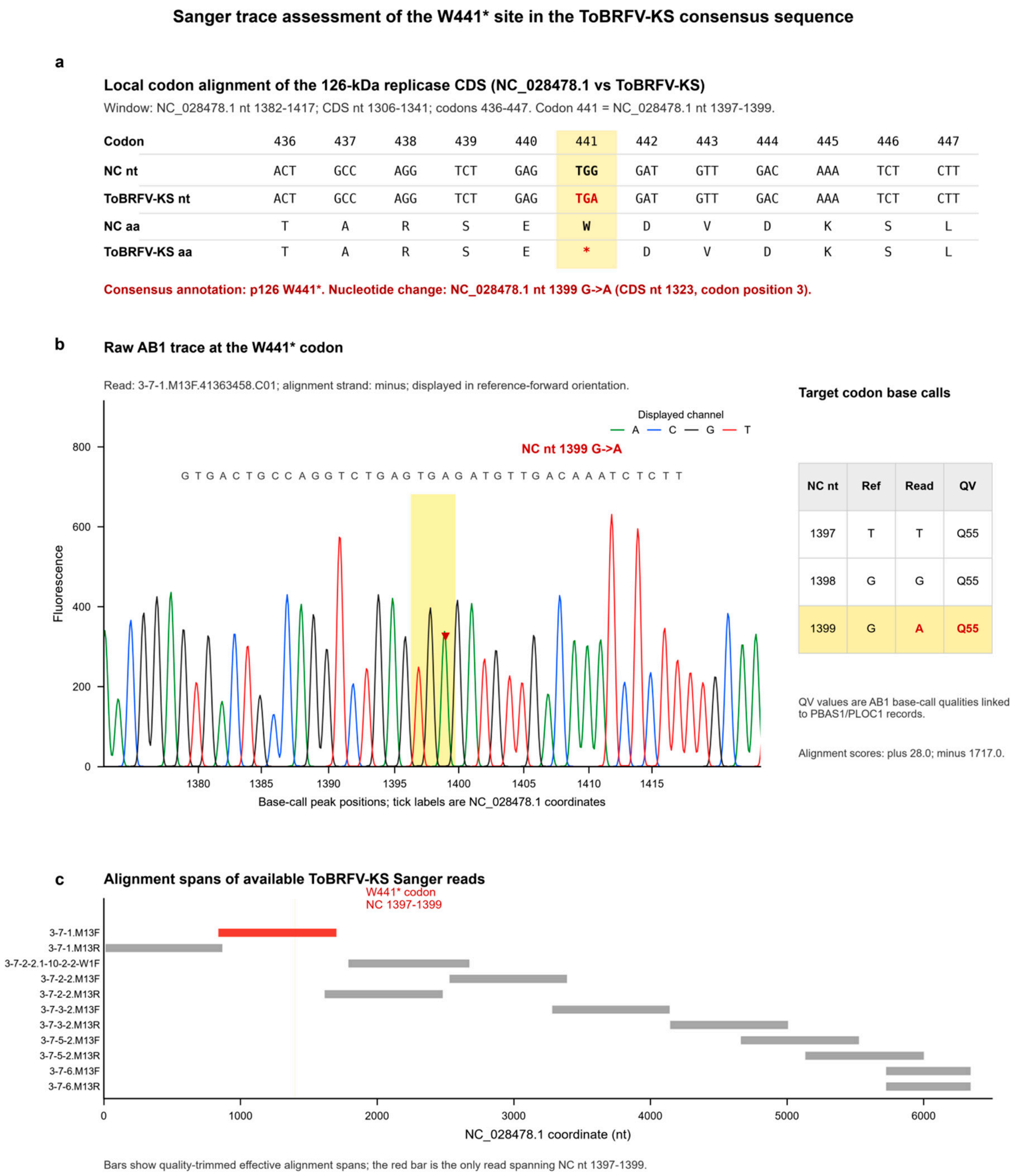

Figure S8. Sanger trace assessment of the ToBRFV-KS p126 W441* site. (a) Codon-aligned comparison of NC_028478.1 and the ToBRFV-KS consensus across NC_028478.1 nt 1382-1417 (126-kDa replicase CDS nt 1306-1341; codons 436-447). Codon 441 corresponds to NC_028478.1 nt 1397-1399; the ToBRFV-KS consensus changes TGG (W) to TGA (*). (b) Raw AB1 chromatogram for read 3-7-1.M13F.41363458.C01. The file name is the original read identity; the read aligns to the minus strand and is displayed in reference-forward orientation with base calls, channel identities and base-call QV values transformed together. (c) Quality-trimmed effective alignment spans of the available ToBRFV-KS Sanger reads on NC_028478.1 coordinates. The coverage overview does not replace the local read-consensus base-call evidence.

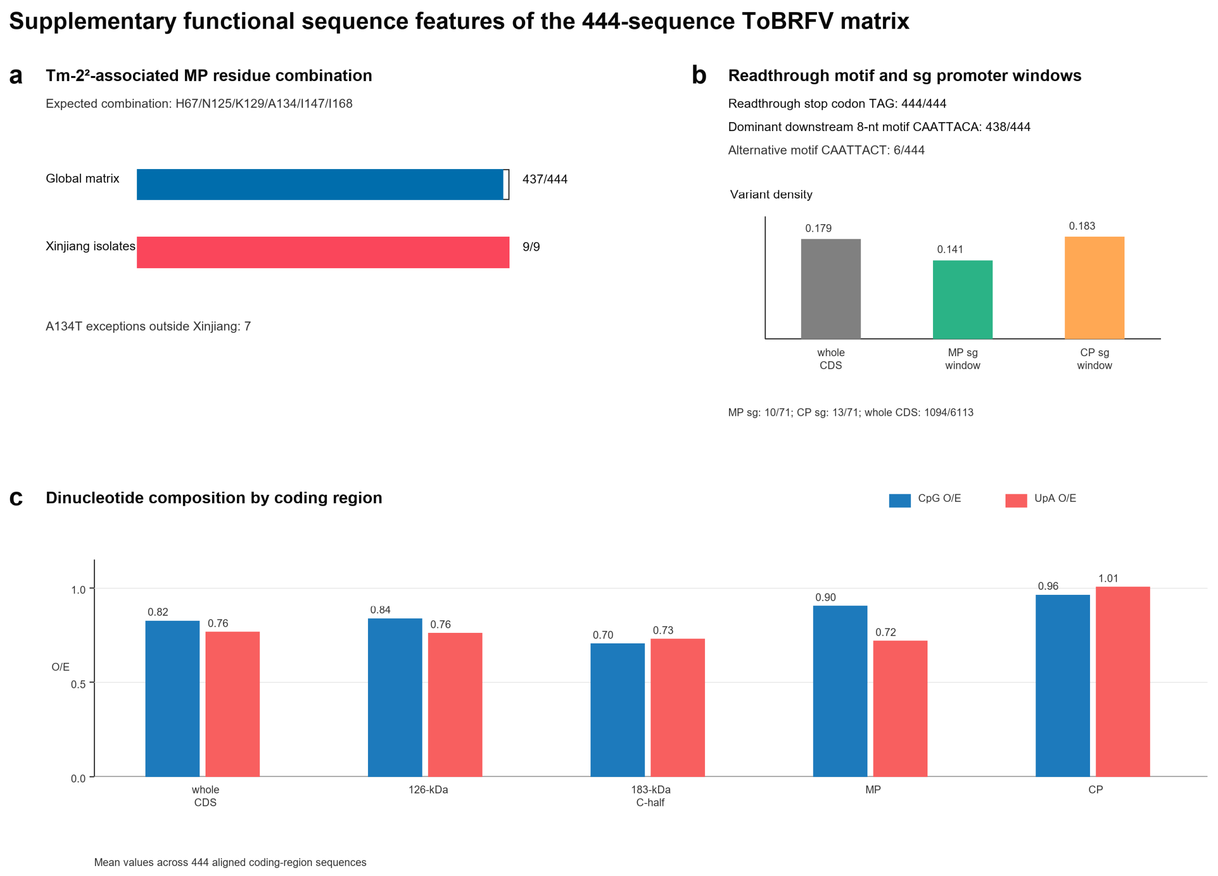

Figure S9. Sequence-based annotation of selected features in the curated 444-sequence ToBRFV coding-region matrix. (a) Frequency of the MP residue combination H67/N125/K129/A134/I147/I168 in the global matrix and in the nine Xinjiang isolates. (b) Conservation of the 126-kDa readthrough stop codon, dominant downstream motif and variant density in 71-position putative MP- and CP-proximal sequence windows defined in the 6,129-nt reference coding projection. (c) Mean CpG and UpA observed/expected ratios by coding region. The figure summarizes sequence-state annotations; resistance phenotype, promoter activity, expression, host-associated biological effects and fitness require functional assays.

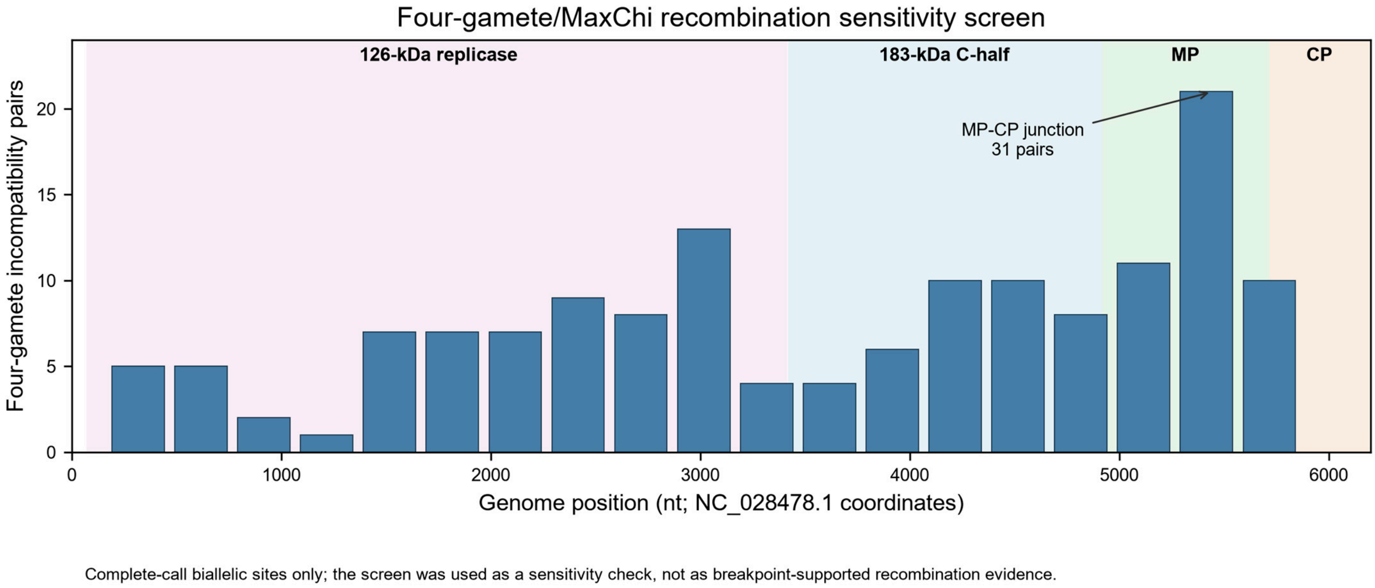

Figure S10. Four-gamete/MaxChi recombination sensitivity screen. The MP-CP junction-localized signal was retained as a sensitivity flag rather than breakpoint-supported recombination evidence.
