# Supplementary material for: Virome Profiling via sRNA-Seq and Sample-Level Coding-Region Diversity of Tomato Brown Rugose Fruit Virus in Protected Tomato Crops in Xinjiang
Source: Viruses. 2026 Oct 11;18(10):1127. doi: 10.3390/v18101127 (PMC13709767; doi:10.3390/v18101127)
Supplement: Supplementary file 1 [file viruses-18-01127-s001.zip › Supplementary_Methods_S1-S3_20260919.pdf]

Supplementary Methods

Methods S1. Coding-region alignment and dataset construction

Public records and reference projection

ToBRFV nucleotide records were downloaded from GenBank on 26 August 2026. Records were retained when their annotated coding features spanned the complete replicase readthrough, movement protein (MP) and coat protein (CP) regions. Patent sequences, UNVERIFIED records, metagenome-derived records and sequences containing IUPAC ambiguity characters were excluded. Country, collection year and host were obtained from GenBank source annotations or local sample records; missing values were recorded as unknown. Accession-level inclusion, exclusion and duplicate-resolution decisions, including included-matrix and exclusion-category summaries, are documented in Supplementary Data S1.

The 435 publicly available sequences and nine Xinjiang genomes were projected to the coding coordinates of reference NC_028478.1. The fixed 6,129-nt alignment concatenated the 183-kDa replicase region (4,848 nt), MP (801 nt) and CP (480 nt). Reference-relative insertion columns were excluded from this fixed-coordinate alignment for phylogenetic, distance and DnaSP analyses. Insertion events were retained in the variant table and region-specific quality-control summaries; deletions were represented as gaps. Complete-call positions contained A, C, G or T in every sequence of the relevant alignment. Pairwise distances instead used positions callable in both members of each sequence pair, as defined in Methods S2.

Three records with annotated open reading frame (ORF) offsets, MW314123.1, OM515264.1 and PP099923.1, were represented by curated ORF extracts before projection. Variants were annotated relative to NC_028478.1 and summarized by coding region. For descriptive variant maps, substitutions were classified by their effect on the corresponding reference codon. ORF-disrupting indels or premature stops were reviewed against the corresponding Xinjiang chromatograms or public-sequence alignment context before use in protein- or codon-based analyses.

Exact sequence types and contextual panel selection

Exact aligned coding-sequence types were defined by identity across the full gap-aware 6,129-nt alignment. Sequence hashes were used only to identify identical aligned strings. Analyses retained individual records, with one representative per exact sequence type used only for methods that required collapsed inputs.

The primary reduced contextual panel retained all nine Xinjiang genomes, all other publicly available Chinese sequences and the earliest public reference sequence. Selection was stratified by country and collection year; exact sequence type was used only for duplicate tracking. For each stratum, the additional quota equalled max(0, k minus the number of forced records), and forced records were retained before quota filling. Strata with records at or below the quota were included in full. In over-represented strata, representative selection was initialized with a most-distant available pair and continued by choosing the candidate with the largest minimum p-distance to the representatives already selected within that stratum. During iterative addition, one-nucleotide-resolution distance ties were resolved by host/date metadata completeness and seeded random selection. A Chinese-background sensitivity analysis perturbed only predefined non-key, exchangeable Chinese public background records by country-year stratum and did not delete Xinjiang genomes, event-defining Chinese anchors or non-Chinese reduced-panel background records.

Candidate caps were k = 4, 6, 8, 10, 12, 14, 16, 18, 20, 24, 28, 32, 36 and 40. Two retention measures were evaluated against the full 444-sequence dataset.

$$R_{PD} = \frac{L_{selected}}{L_{full}} R_S = \frac{S_{panel}}{S_{full}}$$

Here, L_{selected} is the length of the minimal subtree connecting the selected tips, L_{full} is the full-tree length, S_{panel} is the number of segregating sites in the selected panel and S_{full} is the corresponding number in the full matrix. The smallest tested cap satisfying both $R_{\text{PD}} \geq 0.95$ and $R_{\text{S}} \geq 0.95$ defined the primary panel. The primary-panel seed was 20260824, and the 100 replicate-panel seeds were the consecutive integers from 20260825 to 20260924.

Recombination core alignment

The 85-sequence recombination core combined reduced-panel-derived contextual sequences with the predefined reference records specified in the core manifest. The contextual selection retained the Xinjiang and other Chinese sequences, selected Dutch representatives and one representative per other country. Columns containing a gap or a non-ACGT state in any core sequence were removed. Core coordinates refer to the resulting gap-free alignment. Recombination tests and signal-interpretation rules are described in Section 2.8.

Raw small-RNA sequencing data have been deposited in the China National Center for Bioinformation (CNCB) under BioProject PRJCA074566.

Methods S2. Phylogenetic analyses and genetic diversity

Tree inference and placement stability

Maximum-likelihood trees were inferred from the full coding-region matrix, the primary reduced contextual panel and the 100 replicate reduced contextual panels using IQ-TREE v3.0.1. ModelFinder selected substitution models by the Bayesian information criterion. Settings were -m MFP -B 1000 -alrt 1000 -bnni -T AUTO, with seed 20260824 for the primary analysis and the replicate seeds specified in Methods S1. Branch support was assessed using 1,000 ultrafast bootstrap and 1,000 SH-like approximate likelihood-ratio test replicates.

Predefined Xinjiang-placement events were scored across the 100 replicate reduced-panel trees. Event-retention rates of at least 90%, 70-89% and below 70% were classified as stable, conditional and unstable, respectively. The Chinese-background sensitivity analysis applied the same event-scoring rules to the predefined perturbation panels described in Methods S1; its boundary is that event-defining Chinese anchors, all Xinjiang genomes and non-Chinese reduced-panel background records were retained. Reduced-panel and Chinese-background event-retention summaries are provided in Supplementary Table S7. Temporal-signal diagnostics used regression of root-to-tip distance from the midpoint-rooted primary reduced-panel tree against decimal collection year. Exact dates were used when available; year-only records were assigned to mid-year. The five dated-tip filtering strategies and their diagnostic summaries are provided in Supplementary Table S8.

Pairwise distances and standardized public subsampling

For each sequence pair, p-distance was calculated as the number of nucleotide differences divided by the number of positions callable in both sequences. Callable positions contained A, C, G or T in both sequences, so the denominator was determined separately for each pair.

Public comparison groups were drawn from the primary reduced contextual panel after excluding the nine Xinjiang genomes. Each country group with at least eight available records was sampled 1,000 times, selecting eight records without replacement within each draw (seed 20260824). All nine Xinjiang genomes were retained in every comparison. A descriptive between-group distance contrast was calculated for each draw to compare the Xinjiang set with equally sized public subsamples; it was not interpreted as a population differentiation estimate and did not assume that the nine Xinjiang genomes represent the regional virus population.

$$D_{contrast} = 1 - \frac{d_{XJ} + d_{public}}{2d_{XY}}$$

The terms dXJ and dpublic denote mean distances over distinct within-group pairs, and dXY denotes the mean distance over all between-group pairs. A zero dXY yielded an undefined value. Within-group mean distances and the descriptive contrast values were summarized by their medians and empirical 2.5th–97.5th percentiles across draws. These intervals describe the standardized subsampling distribution. Public draws used eight records; all nine Xinjiang genomes were retained in every comparison.

Sequence type networks and ordination

Relationships among exact coding-sequence types were displayed as minimum-spanning networks generated with NetworkX (spring-layout seed 42). Principal-coordinate analysis (PCoA) used the full 444 × 444 pairwise p-distance matrix. Squared distances were double-centred as follows.

$$B = -\frac{1}{2}JD^2J \quad J = I - \frac{11^T}{n}$$

Here, D is the pairwise distance matrix, I is the identity matrix, 1 is an all-ones column vector and n is the number of sequences. Eigen decomposition was performed in NumPy. Coordinates were obtained by multiplying the leading eigenvectors by the square roots of their positive eigenvalues, and axis percentages were calculated relative to the sum of positive eigenvalues. The distance and ordination procedures are documented in the accompanying scripts and generated matrix outputs.

DnaSP summaries and sliding windows

DnaSP v6.12.01 [15] was run with FASTA/DNA/haploid input, All Included Sequences and positions 1–6,129. The full 444-record matrix, primary reduced contextual panel and nine-sequence Xinjiang alignment retained individual sequence records, including identical sequences, rather than pre-collapsed representatives. Columns containing gaps or missing data were excluded within each analysed alignment; the resulting common-site denominator can therefore differ from the pair-specific distance denominator.

Statistics comprised nucleotide diversity (π), segregating sites (S), total mutations (Eta), haplotype number (h), haplotype diversity (Hd), Watterson's θ per site and DnaSP mean pairwise differences (k). Tajima's D, Fu's Fs, Fu and Li's D* and F*, and Achaz's Y* were obtained for the full matrix, the reduced contextual panel and eligible public groups with $n \geq 20$. For the nine Xinjiang genomes, the same diversity estimates were summarized descriptively as sample-level statistics; no neutrality, regional frequency or population-diversity inference was made from this nine-genome set. Fu and Li's and Achaz's summaries followed the software's applicable biallelic-site handling.

Sliding-window π was calculated separately with custom scripts using 300-nt windows and a 100-nt step for

Xinjiang, other Chinese sequences in the reduced panel, the Netherlands subset in the reduced panel and the reduced public background. These calculations support the diversity summary and sliding-window profiles reported in Fig. 4.

Clade-marker cross-reference and MP260I verification

Clade-defining markers from the NPPO-NL ToBRFV Nextstrain/Nextclade dataset v5 (updated 4 August 2025; coding-sequence reference KT383474.1) were mapped to the fixed 6,129-nt coding-region alignment. A clade label was assigned only when all mapped nucleotide markers for that clade were present. Xinjiang genomes matching the clade_7 nucleotide-marker profile were additionally checked against the MP260I protein marker. These labels were generated by marker-rule matching rather than a formal Nextclade CLI run.

ToBRFV-YL, ToBRFV-TLF1, ToBRFV-TLF2 and ToBRFV-KZ matched all three clade_7 nucleotide markers and passed the MP260I check. ToBRFV-XJ, ToBRFV-AKS, ToBRFV-KS, ToBRFV-HT1 and ToBRFV-HT2 matched none of the three clade_7 nucleotide markers, did not carry the MP260I marker and did not satisfy the complete nucleotide-marker set for any other clade; they were therefore retained as unassigned. These results provide a descriptive cross-reference to the phylogenetic groupings reported in Section 3.2.

Accession-level clade labels for the 444-sequence dataset, comparison with the NPPO-NL reference-tree labels, and the nucleotide-marker and MP260I checks for the nine Xinjiang genomes are provided in Supplementary Data S2. The clade-marker check and the codon-model site analyses in Section 3.4 answer different questions; agreement with a clade-marker profile was used only for sequence annotation and nomenclature cross-reference.

Methods S3. Codon models and sensitivity analyses

Regional inputs and user trees

Region-specific codon alignments were prepared from the primary reduced contextual panel for *CP* (480 nt), *MP* (801 nt), the 126-kDa replicase region (3,351 nt) and the 183-kDa readthrough replicase coding region (4,848 nt). These lengths refer to the extracted alignments before codon-column filtering and include the corresponding stop codons. Sequences containing ambiguous codons or unexpected ORF-disrupting changes were excluded from the relevant regional analysis; canonical terminal stop codons and the conserved readthrough site were handled separately as described below. ToBRFV-KS was excluded from both replicase analyses because of the W441* premature stop in their shared N-terminal region. Identical regional codon sequences were collapsed to one representative for the primary codeml analyses, with accession maps linking representatives to the original records.

In PAML v4.10.10, codon columns containing stop codons were masked as missing data and subsequently removed with cleandata = 1. For the 183-kDa alignment, removal of the conserved TAG readthrough column (regional nucleotide positions 3,349–3,351; codon 1,117) and the terminal TAA column (positions 4,846–4,848; codon 1,616) left 1,614 codons for model fitting. Removal of the terminal stop-codon column from the 126-kDa, *MP* and *CP* alignments left 1,116, 266 and 159 codons, respectively.

Site models were fitted using codeml in PAML v4.10.10 [17]. Region-specific neighbour-joining user trees were constructed from identity distances using Bio.Phylo. Tree topology was held fixed while branch lengths were estimated. Control settings were runmode = 0, seqtype = 1, CodonFreq = 2, cleandata = 1, kappa = 2 and omega = 0.4; the NSsites values were 0, 1, 2, 7 and 8.

Model comparisons and site reporting

M1a–M2a and M7–M8 likelihood-ratio tests used a chi-square reference distribution with two degrees of freedom. Sites with Bayes empirical Bayes (BEB) posterior probabilities ≥0.95 were reported as sequence-based candidates when the corresponding likelihood-ratio test supported the model allowing $\omega > 1$. BEB posterior probabilities and fixed-effects likelihood (FEL) *P* values were treated as distinct evidence measures. Because the 126-kDa coding region is nested within the 183-kDa readthrough replicase coding region, support for shared candidate codons in the two analyses was not treated as statistically independent evidence.

Raw BEB site numbers refer to the cleaned codon alignments. All replicase candidate sites reported here precede the excluded internal readthrough column and therefore retain the same numbering as in the original regional alignments. Amino acid labels in raw BEB output refer to the first sequence in the corresponding input alignment.

Duplicate handling and numerical sensitivity

The primary *CP* and *MP* analyses used 80 and 147 exact regional sequence types, corresponding to 300 and 299 eligible records, respectively. Each replicase dataset contained 299 eligible records, which were collapsed to 254 exact regional sequence types for the 126-kDa region and 265 for the 183-kDa region.

The *CP* sensitivity analysis retained all eligible records and used the same codeml model framework with freely estimated branch lengths. For *MP*, the duplicate-retaining codeml analysis failed to converge with freely estimated branch lengths; an all-eligible-record FEL analysis in HyPhy v2.5.101 [31] was therefore used as a sensitivity check. This comparison evaluates the combined effects of duplicate handling and model choice and does not distinguish their individual contributions.

No final duplicate-retaining codeml or FEL sensitivity comparison was completed for either replicase region. Candidate-site inferences from these regions were therefore retained as exploratory sequence-based evidence.

Diagnostic runs with fixed branch lengths yielded markedly different *CP* likelihood-ratio results from runs with freely estimated branch lengths. Inference was therefore based on codeml runs with freely estimated branch lengths and, for the all-eligible-record *MP* sensitivity analysis, the FEL analysis described above. Only region–model combinations with complete final likelihood and parameter outputs were used for inference. Input counts, exclusions, model summaries and sensitivity results are provided in Supplementary Table S10; Figure 6 shows the genomic positions and support classes of the candidate residues.
